# A Body Shape Index (ABSI) achieves better mortality risk stratification than alternative indices of abdominal obesity: results from a large European cohort

**DOI:** 10.1038/s41598-020-71302-5

**Published:** 2020-09-03

**Authors:** Sofia Christakoudi, Konstantinos K. Tsilidis, David C. Muller, Heinz Freisling, Elisabete Weiderpass, Kim Overvad, Stefan Söderberg, Christel Häggström, Tobias Pischon, Christina C. Dahm, Jie Zhang, Anne Tjønneland, Jytte Halkjær, Conor MacDonald, Marie-Christine Boutron-Ruault, Francesca Romana Mancini, Tilman Kühn, Rudolf Kaaks, Matthias B. Schulze, Antonia Trichopoulou, Anna Karakatsani, Eleni Peppa, Giovanna Masala, Valeria Pala, Salvatore Panico, Rosario Tumino, Carlotta Sacerdote, J. Ramón Quirós, Antonio Agudo, Maria-Jose Sánchez, Lluís Cirera, Aurelio Barricarte-Gurrea, Pilar Amiano, Ensieh Memarian, Emily Sonestedt, Bas Bueno-de-Mesquita, Anne M. May, Kay-Tee Khaw, Nicholas J. Wareham, Tammy Y. N. Tong, Inge Huybrechts, Hwayoung Noh, Elom K. Aglago, Merete Ellingjord-Dale, Heather A. Ward, Dagfinn Aune, Elio Riboli

**Affiliations:** 1 Department of Epidemiology and Biostatistics, Imperial College London, Norfolk Place, St Mary’s Campus, London, W2 1PG UK; 2 MRC Centre for Transplantation, King’s College London, Great Maze Pond, London, SE1 9RT UK; 3 Department of Hygiene and Epidemiology, School of Medicine, University of Ioannina, 45100 Ioannina, Greece; 4 International Agency for Research on Cancer, World Health Organization, 150 Cours Albert Thomas, 69372 Lyon CEDEX 08, France; 5 Department of Public Health, Aarhus University, DK-8000 Aarhus, Denmark; 6 Department of Cardiology, Aalborg University Hospital, DK-9000 Aalborg, Denmark; 7 Department of Public Health and Clinical Medicine, Umeå University, Umeå, Sweden; 8 Department of Biobank Research, Umeå University, Umeå, Sweden; 9 Department of Surgical Sciences, Uppsala University, Uppsala, Sweden; 10 Max Delbrück Center for Molecular Medicine, 13125 Berlin, Germany; 11 Charité – Universitätsmedizin Berlin, Berlin, Germany; 12 Berlin Institute of Health (BIH), Berlin, Germany; 13 Danish Cancer Society Research Center, Strandboulevarden 49, DK-2100 Copenhagen Ø, Denmark; 14 Department of Public Health, University of Copenhagen, Copenhagen, Denmark; 15 Centre de recherche en Epidemiologie et Sante des Populations (CESP), Fac. de médecine - Univ. Paris-Sud, Fac. de médecine - UVSQ, INSERM, Université Paris-Saclay, 94805 Villejuif, France; 16 Gustave Roussy, F-94805 Villejuif, France; 17 German Cancer Research Center (DKFZ), Division of Cancer Epidemiology, Heidelberg, Germany; 18 Department of Molecular Epidemiology, German Institute of Human Nutrition Potsdam-Rehbruecke, Nuthetal, Germany; 19 Institute of Nutritional Sciences, University of Potsdam, Nuthetal, Germany; 20 Hellenic Health Foundation, Athens, Greece; 21 2nd Pulmonary Medicine Department, School of Medicine, National and Kapodistrian University of Athens, “ATTIKON” University Hospital, Haidari, Greece; 22 Cancer Risk Factors and Life-Style Epidemiology Unit, Institute for Cancer Research, Prevention and Clinical Network - ISPRO, Florence, Italy; 23 Epidemiology and Prevention Unit, Fondazione IRCCS Istituto Nazionale dei Tumori di Milano, Via Venezian, 1, 20133 Milan, Italy; 24 Dipartimento di Medicina Clinica e Chirurgia, Federico II University, Naples, Italy; 25 Cancer Registry and Histopathology Department, Azienda Sanitaria Provinciale Ragusa (ASP), Ragusa, Italy; 26 Unit of Cancer Epidemiology, Città della Salute e della Scienza University-Hospital and Center for Cancer Prevention (CPO), Turin, Italy; 27 Public Health Directorate, Asturias, Spain; 28 Unit of Nutrition and Cancer, Cancer Epidemiology Research Program, Catalan Institute of Oncology-IDIBELL, L’Hospitalet de Llobregat, Barcelona, Spain; 29 Andalusian School of Public Health (EASP), Granada, Spain; 30 Instituto de Investigación Biosanitaria de Granada ibs.GRANADA, Granada, Spain; 31 Universidad de Granada, Granada, Spain; 32 CIBER Epidemiology and Public Health (CIBERESP), Madrid, Spain; 33 Department of Epidemiology, Murcia Regional Health Council, IMIB - Arrixaca, Murcia, Spain; 34 Department of Health and Social Sciences, University of Murcia, Murcia, Spain; 35 Navarra Public Health Institute, Pamplona, Spain; 36 Navarra Institute for Health Research (IdiSNA) Pamplona, Pamplona, Spain; 37 Public Health Division of Gipuzkoa, BioDonostia Research Institute, San Sebastian, Spain; 38 Dept. Clinical Sciences, Skane University Hospital, Lund University, 20502 Malmö, Sweden; 39 Nutritional Epidemiology, Department of Clinical Sciences Malmö, Lund University, Malmö, Sweden; 40 Department for Determinants of Chronic Diseases (DCD), National Institute for Public Health and the Environment (RIVM), P.O. Box 1, 3720 BA Bilthoven, The Netherlands; 41 Department of Gastroenterology and Hepatology, University Medical Centre, Utrecht, The Netherlands; 42 Department of Social and Preventive Medicine, Faculty of Medicine, University of Malaya, Pantai Valley, 50603 Kuala Lumpur, Malaysia; 43 Julius Center for Health Sciences and Primary Care, University Medical Center Utrecht, Utrecht University, STR 6.131, P.O. Box 85500, 3508 GA Utrecht, The Netherlands; 44 School of Clinical Medicine, Addenbrooke’s Hospital, University of Cambridge, Cambridge, CB2 2QQ UK; 45 MRC Epidemiology Unit, Institute of Metabolic Science, School of Clinical Medicine, University of Cambridge, Cambridge, CB2 0QQ UK; 46 Cancer Epidemiology Unit, Nuffield Department of Population Health, University of Oxford, Oxford, UK; 47 Department of Nutrition, Bjørknes University College, Oslo, Norway; 48 Department of Endocrinology, Morbid Obesity and Preventive Medicine, Oslo University Hospital, Oslo, Norway

**Keywords:** Risk factors, Metabolic disorders, Obesity, Weight management, Lifestyle modification

## Abstract

Abdominal and general adiposity are independently associated with mortality, but there is no consensus on how best to assess abdominal adiposity. We compared the ability of alternative waist indices to complement body mass index (BMI) when assessing all-cause mortality. We used data from 352,985 participants in the European Prospective Investigation into Cancer and Nutrition (EPIC) and Cox proportional hazards models adjusted for other risk factors. During a mean follow-up of 16.1 years, 38,178 participants died. Combining in one model BMI and a strongly correlated waist index altered the association patterns with mortality, to a predominantly negative association for BMI and a stronger positive association for the waist index, while combining BMI with the uncorrelated A Body Shape Index (ABSI) preserved the association patterns. Sex-specific cohort-wide quartiles of waist indices correlated with BMI could not separate high-risk from low-risk individuals within underweight (BMI < 18.5 kg/m^2^) or obese (BMI ≥ 30 kg/m^2^) categories, while the highest quartile of ABSI separated 18–39% of the individuals within each BMI category, which had 22–55% higher risk of death. In conclusion, only a waist index independent of BMI by design, such as ABSI, complements BMI and enables efficient risk stratification, which could facilitate personalisation of screening, treatment and monitoring.

## Introduction

Obesity contributes to premature death^1^ but specific fat locations are differentially associated with the outcomes of obesity, with the metabolic complications of obesity associated positively with abdominal adiposity and negatively with gluteofemoral adiposity^2^. Correspondingly, individuals with normal-weight and abdominal obesity can show metabolic alterations and, hence, a higher risk of death, while obese individuals without abdominal adiposity can remain “metabolically healthy”^3–6^. The European Prospective Investigation into Cancer and Nutrition (EPIC) was the first large study to confirm that both abdominal and general adiposity are independently associated with the risk of death and to recommend using a waist index in addition to BMI^7^.

Nevertheless, while general obesity is widely evaluated with body mass index (BMI)^8^, according to the well-known World Health Organisation (WHO) categories^9^, there is no current consensus on how best to assess abdominal adiposity and various anthropometric indices incorporating waist circumference (WC) have been proposed in the literature^10–16^. A major problem when assessing abdominal adiposity stems from the strong correlation between BMI and WC^17^. This hinders risk stratification within underweight or obese (BMI ≥ 35 kg/m^2^) categories, when BMI is combined with WC or the waist-to-hip ratio (WHR)^18^, and precludes personalisation of screening and clinical management^19^. To account for the correlation with BMI, separate cut-offs for WC have been proposed for individual BMI categories^20^ and genetic studies have used residuals of WC or WHR adjusted for BMI^21^. A Body Shape Index (ABSI), however, was specifically designed as independent from BMI^22^. ABSI is based on the allometric principle^23, 24^, previously used for the development of BMI^25^ (see Supplementary Note online), and is positively associated with all-cause mortality^22, 26–33^. In analogy to ABSI, an allometric Hip Index (HI) was developed as an independent of BMI alternative to hip circumference (HC), the traditional measure of gluteofemoral adiposity^34^.

To provide clarity about the usefulness of various body shape indices when assessing the risk of death, we compared systematically combinations of traditional or non-traditional body shape indices with BMI, using data from the large EPIC cohort, which has accumulated seven years longer follow-up and double the number of deaths since the original report^7^. Our aim was to determine the most appropriate body shape index, which can provide additional information to BMI and can enable risk stratification, i.e. separation into high-risk and low-risk subgroups, within each WHO category of BMI.

## Methods

### Study population

The EPIC cohort and data accrual have previously been described^7, 35–37^. Supplementary Fig. S1 online shows a flow diagram of individuals included in the current study, with sequential exclusions related to data availability and quality.

### Endpoint

The outcome was death from all causes. Cause-specific analyses were beyond the scope of this study. Vital status and the date of death were ascertained via record linkage to cancer or death registries or by active follow-up, including enquiries to municipal registries, physicians, hospitals, or next of kin^7^.

### Anthropometric indices

Anthropometric measurements were obtained by trained personnel and were systematically adjusted for clothing, as previously described^7, 35^. Individuals with self-reported values were excluded. In the main analyses, we used BMI (as an index of general adiposity); ABSI, WC and WHR (as indices of abdominal adiposity); HC and HI (as indices of gluteofemoral adiposity). We additionally examined for comparison alternative WC-based anthropometric indices. The calculation of anthropometric indices is described below, with the relevant reference (ref) cited at the end of each formula:

- ABSI (A Body Shape Index) = 1,000*WC*Wt ^–2/3^*Ht^5/6^ ref^22^
- AVI (Abdominal Volume Index) = (2*(WC*100)^2^ + 0.7*(WC*100 − HC*100)^2^)/1,000 ref^11^
- BMI (Body Mass Index) = Wt/Ht^2^
- BRI (Body Roundness Index) = 364.2–365.5*(1 − ((0.5*WC/π)^2^/(0.5*Ht)^2^))^0.5^ ref^14^
- ConI (Conicity Index) = WC/(0.109*(Wt/Ht)^0.5^) ref^15^
- eTBF (estimated Total Body Fat) = 100 * (–Z + A − B)/C, where A = (4.15*WC*39.3701), B = (0.082*Wt*2.20462), C = (Wt*2.20462), Z = 98.42 (men), Z = 76.76 (women) ref^12^
- RFM (Relative Fat Mass) = 64 − (20*Ht/WC) + (12*S), where S = 0 (men), S = 1 (women) ref^16^
- HI (Hip Index) = HC * Wt ^–0.482^*Ht^0.310^ ref^34^
- WHR (Waist-to-Hip Ratio) = WC/HC
- WHtR (Waist-to-Height Ratio) = WC/Ht
- WWI (Weight-adjusted Waist Index) = (WC*100)/(Wt^0.5^) ref^13^
- WCadjBMI (WC adjusted for BMI) and WHRadjBMI (WHR adjusted for BMI) were derived as the residuals of sex-specific linear regression models WC (or WHR) ~ BMI + study centre.

HC—hip circumference (m); WC—waist circumference (m); Ht—height (m); Wt—weight (kg). ABSI was multiplied by 1,000 to derive numbers in the order of magnitude of WC, which would be more intuitive to use than the original values, which are < 0.1. The formula for eTBF incorporates factors to convert the measurements into units matching the original formula: 39.3701 for a conversion from m to in and 2.20462 from kg to lbs.

### Statistical analysis

We examined men and women separately. We summarised continuous variables with mean (standard deviation, SD) and categorical variables with percentages of individuals per category. We assessed associations between obesity indices with partial Pearson correlation coefficients (r), adjusted for age at recruitment and study centre. We additionally examined the association of body shape indices with BMI in linear regression models adjusted for age at recruitment and study centre, using for each body shape index sex-specific z-scores calculated as (index—mean)/SD. Using z-scores on an SD scale enabled comparisons between obesity indices measured with different units. Using a 5 kg/m^2^ increment for BMI approximated the difference in BMI between neighbouring WHO categories of BMI, such that the Wald tests from these models evaluated a linear trend by BMI category.

We compared body shape indices in three steps, as described below:

First, we examined changes in the association patterns of individual obesity indices with mortality determined by combining body shape indices with BMI in the same model. In these analyses we used a more detailed categorisation of exposure variables, including sex-specific cohort-wide quintiles for waist and hip indices (see cut-offs in Supplementary Table S1 online) and nine categories for BMI, with cut-offs at 18.5, 21.0, 23.5, 25.0, 26.5, 28.0, 30.0, 35.0 kg/m^2^ (23.5 to < 25 reference)^7^. We used delayed-entry Cox proportional hazards models, stratified by age (5-year intervals) and study centre, and obtained hazard ratio (HR) estimates with 95% confidence intervals. The underlying time scale was age. The origin of time was at birth. Entry in the study was at the age of recruitment and exit was at the age of censoring or death. All models included adjustment for major risk factors for death and potential confounders: smoking status and intensity, attained education level (as the nearest available proxy for socioeconomic status), alcohol consumption, physical activity and height. Covariates were categorised according to the original EPIC publication, with missing data similarly coded as a separate category^7^.

Second, we calculated Kaplan–Meier estimates for 15-year probability of death for subgroups defined by BMI and a waist index, in order to compare the ability of alternative waist indices to achieve risk stratification within individual categories of BMI. In these analyses we used sex-specific cohort-wide quartiles for waist indices and five WHO categories for BMI: < 18.5 (underweight); 18.5 to < 25 (normal-weight: reference); 25 to < 30 (overweight); 30 to < 35 (obese grade I) and ≥ 35 kg/m^2^ (obese grade II and III)^9^.

Third, we compared the ability of the best performing index of abdominal obesity (ABSI) and the traditional indices (WC and WHR) to separate subgroups with low-waist and high-waist within each WHO category of BMI, using published cut-offs for WC and WHR. For WC, we used the WHO cut-offs (102 cm for men; 88 cm for women)^18^ and the BMI-specific cut-offs proposed by Ardern et al*.*^20^. The latter were defined for normal-weight, overweight, obese grade I, and obese grade II and III categories (90, 100, 110, and 125 cm for men; 80, 90, 105, and 115 cm for women). We complemented the missing cut-offs for the underweight category with 10 cm lower values compared to the cut-offs proposed for the normal-weight category (80 cm for men; 70 cm for women). For WHR, we used the WHO cut-offs (0.90 for men, 0.85 for women)^18^. For ABSI we selected the 75th sex-specific cohort-wide centile (83.3 for men; 76.2 for women). We calculated adjusted HRs using Cox proportional hazards models, as described for the first step above, with waist-by-BMI group as exposure variable and low-waist-normal-weight as reference. We additionally calculated HRs for high-waist vs. low-waist within each BMI category (function* glht*, package ***multcomp***)^38^. We used the likelihood ratio test to assess a potential waist-by-BMI interaction on a multiplicative scale (function ***lrtest,*** package ***lmtest***)^39^, comparing the cross-classification model (equivalent to a waist-by-BMI interaction model), with a model including the waist index and BMI as individual variables.

Finally, we examined heterogeneity in the association of ABSI and BMI with mortality according to categories of other risk factors. We created a combined cross-classification variable using ABSI-by-BMI and three categories for each of the common risk factors for death and obesity: smoking status, physical activity, age at recruitment or attained education. We defined the survival models as for the cross-classification with ABSI-by-BMI but omitted the examined risk factor from the adjustment or stratification. The likelihood ratio test for statistical interaction compared the cross-classification model with a model including ABSI-by-BMI categories and the risk factor as separate variables.

We used R version 3.4.3 for all statistical analyses^40^.

### Ethical approval and consent to participate

This research was conducted according to the principles expressed in the Declaration of Helsinki. Approval for the EPIC study was obtained from the ethical review boards of the International Agency for Research on Cancer and from all participating EPIC centres. All EPIC participants provided written informed consent at recruitment for use of their blood samples and data in future research. The EPIC Steering Committee approved this study in accordance with EPIC rules https://epic.iarc.fr/access/access_appl_assessed.php.

## Results

### Characteristics of study participants

Cohort characteristics and waist indices are summarised by sex and BMI category in Table 1. There were 38,178 deaths among 352,985 participants (34.3% men), for a mean follow-up of 16.1 years (SD = 3.7). The mean BMI at recruitment was 26.6 (SD = 3.6) kg/m^2^ for men and 25.5 (4.6) kg/m^2^ for women. WHR was above the high-risk WHO cut-offs in 76% of men and only 20% of women. WC was above the WHO cut-offs in 23% of men and women. After accounting for age at recruitment, individuals who died during each year showed consistently higher BMI compared to those who survived by the end of the same year only after the first seven years (see Supplementary Table S3 online).

**Table 1:** Cohort characteristics and body shape indices by sex and BMI categories.

Men							
Cohort	Total	BMI < 18.5	18.5 to < 25	25 to < 30	30 to < 35	BMI ≥ 35	SD per 5 kg/m^2^
Cohort	120,915	451 (0.4)	41,094 (34.0)	59,931 (49.6)	16,744 (13.8)	2,695 (2.2)	–
Deaths	18,636 (15.4)	144 (31.9)	5,979 (14.5)	8,823 (14.7)	2,993 (17.9)	697 (25.9)	–
Follow-up	15.6 (4.1)	14.4 (5.3)	15.8 (4.0)	15.7 (4.1)	15.3 (4.4)	14.5 (4.7)	− 0.08 (0.004)
Age	52.8 (9.6)	50.8 (15.1)	51.4 (10.7)	53.4 (9.0)	53.8 (8.6)	53.8 (8.5)	0.14 (0.004)
BMI	26.6 (3.6)	17.6 (0.8)	23.0 (1.5)	27.2 (1.4)	31.8 (1.3)	37.6 (2.9)	–
**Waist indices**							
ABSI	80.6 (4.2)	82.5 (6.2)	80.1 (4.5)	80.7 (4.1)	81.4 (4.0)	81.7 (4.3)	0.14 (0.004)
AVI	18.2 (3.9)	11.2 (1.7)	14.9 (2.1)	18.6 (2.4)	23.1 (2.8)	29.1 (4.2)	1.18 (0.002)
BRI	4.3 (1.3)	2.0 (0.5)	3.2 (0.7)	4.4 (0.8)	6.0 (0.9)	7.9 (1.3)	1.19 (0.002)
ConI	1.28 (0.08)	1.22 (0.09)	1.24 (0.07)	1.28 (0.07)	1.33 (0.07)	1.37 (0.07)	0.64 (0.003)
eTBF	22.9 (6.3)	10.4 (7.5)	18.4 (5.6)	24.1 (4.9)	28.6 (4.5)	31.7 (4.5)	0.89 (0.003)
RFM	26.7 (4.3)	16.3 (3.6)	22.9 (3.2)	27.7 (2.5)	31.7 (2.0)	35.2 (2.0)	1.16 (0.002)
WC	94.7 (10.2)	74.1 (5.8)	86.0 (6.1)	96.2 (6.2)	107.2 (6.4)	120.2 (8.5)	1.18 (0.002)
WCadjBMI	0 (0.051)	0.01 (0.058)	− 0.0015 (0.049)	0.0010 (0.050)	0.0013 (0.055)	− 0.0074 (0.077)	0 (0.004)
WHR	0.94 (0.06)	0.84 (0.06)	0.90 (0.06)	0.95 (0.05)	0.99 (0.05)	1.02 (0.06)	0.78 (0.003)
WHRadjBMI	0 (0.051)	− 0.003 (0.054)	− 0.0036 (0.05)	0.0034 (0.051)	0.0009 (0.053)	− 0.025 (0.066)	0 (0.004)
WHtR	0.54 (0.06)	0.42 (0.03)	0.49 (0.04)	0.55 (0.04)	0.62 (0.04)	0.70 (0.05)	1.19 (0.002)
WWI	10.54 (0.69)	10.04 (0.78)	10.19 (0.63)	10.61 (0.61)	11.04 (0.61)	11.40 (0.65)	0.66 (0.003)
**Hip indices**							
HC	100.9 (6.9)	88.0 (4.6)	95.7 (4.6)	101.6 (4.7)	108.4 (5.2)	117.8 (8.1)	1.06 (0.003)
HI	0.145 (0.006)	0.153 (0.008)	0.146 (0.006)	0.144 (0.005)	0.143 (0.006)	0.144 (0.008)	− 0.26 (0.004)

Women							
Cohort	Total	BMI < 18.5	18.5 to < 25	25 to < 30	30 to < 35	BMI ≥ 35	SD per 5 kg/m^2^
Cohort	232,070	3,967 (1.7)	119,270 (51.4)	73,515 (31.7)	26,181 (11.3)	9,137 (3.9)	–
Deaths	19,542 (8.4)	396 (10.0)	8,530 (7.2)	6,733 (9.2)	2,720 (10.4)	1,163 (12.7)	–
Follow-up	16.4 (3.5)	16.8 (3.6)	16.6 (3.3)	16.2 (3.5)	15.8 (3.8)	15.4 (4.0)	− 0.11 (0.002)
Age	51.2 (10.5)	46.0 (12.9)	49.3 (10.8)	53.2 (9.6)	54.2 (9.2)	53.8 (9.1)	0.23 (0.002)
BMI	25.5 (4.6)	17.7 (0.7)	22.3 (1.6)	27.1 (1.4)	32.0 (1.4)	38.4 (3.4)	–
**Waist indices**							
ABSI	73.1 (5.2)	74.3 (5.0)	72.3 (4.9)	73.5 (5.4)	74.5 (5.6)	74.4 (5.8)	0.14 (0.002)
AVI	13.5 (3.8)	8.7 (1.1)	11.2 (1.8)	14.5 (2.3)	18.3 (2.8)	23.0 (4.1)	0.94 (0.001)
BRI	3.4 (1.5)	1.6 (0.4)	2.5 (0.7)	3.9 (0.9)	5.4 (1.2)	7.3 (1.6)	0.94 (0.001)
ConI	1.15 (0.09)	1.10 (0.07)	1.11 (0.08)	1.17 (0.09)	1.22 (0.09)	1.25 (0.10)	0.51 (0.002)
eTBF	28.2 (8.3)	19.2 (6.7)	24.4 (6.7)	31.1 (7.0)	35.6 (7.1)	37.7 (6.9)	0.63 (0.002)
RFM	34.9 (5.9)	25.0 (3.4)	31.1 (3.9)	37.5 (3.4)	42.2 (3.0)	46.0 (3.0)	0.92 (0.001)
WC	80.2 (11.4)	64.5 (4.4)	73.1 (6.2)	84.1 (7.0)	94.6 (7.7)	106.2 (9.8)	0.93 (0.001)
WCadjBMI	0 (0.057)	0.011 (0.045)	− 0.0026 (0.049)	0.0029 (0.059)	0.0058 (0.068)	− 0.011 (0.086)	0 (0.002)
WHR	0.79 (0.07)	0.74 (0.05)	0.77 (0.06)	0.81 (0.07)	0.84 (0.07)	0.86 (0.07)	0.50 (0.002)
WHRadjBMI	0 (0.06)	0.0061 (0.054)	− 0.0034 (0.056)	0.0056 (0.063)	0.0064 (0.066)	− 0.022 (0.07)	0 (0.002)
WHtR	0.50 (0.08)	0.39 (0.03)	0.45 (0.04)	0.52 (0.05)	0.60 (0.05)	0.67 (0.06)	0.94 (0.001)
WWI	9.85 (0.86)	9.37 (0.66)	9.51 (0.70)	10.06 (0.80)	10.54 (0.85)	10.87 (0.90)	0.54 (0.002)
**Hip indices**							
HC	101.1 (9.3)	87.2 (4.4)	95.6 (5.2)	103.9 (5.3)	112.2 (5.8)	124.0 (8.8)	0.94 (0.001)
HI	0.156 (0.007)	0.158 (0.007)	0.156 (0.006)	0.155 (0.006)	0.156 (0.007)	0.159 (0.008)	0.05 (0.002)

ABSI—A Body Shape Index; AVI—Abdominal Volume Index; BMI—Body Mass Index; ConI—Conicity Index; eTBF—estimated Total Body Fat; HC—Hip Circumference; HI—Hip Index; RFM—Relative Fat Mass; SD—standard deviation; WC—Waist Circumference; WCadjBMI—WC adjusted for BMI; WHR—Waist-to-Hip Ratio; WHRadjBMI—WHR adjusted for BMI; WHtR—Waist-to-Height Ratio; WWI—Weight-adjusted Waist Index; Cohort—number of individuals (% percentage from the total); Deaths—number of deaths (% percentage from the total number of individuals per column); Total/BMI columns—continuous variables are summarised with mean (SD); SD per 5 kg/m^2^—mean body shape index increment on the SD scale (standard error), derived from linear models regressing the sex-specific z-scores of the corresponding body shape index on BMI (per 5 kg/m^2^ increment), with adjustment for age at recruitment and study centre (all *p* values from the corresponding Wald tests were < 0.0001, except for WCadjBMI and WHRadjBMI); Covariates are summarised by sex and BMI category in Supplementary Table S2 online.

### Waist indices formed groups according to the strength of their association with BMI

**Figure 1:**
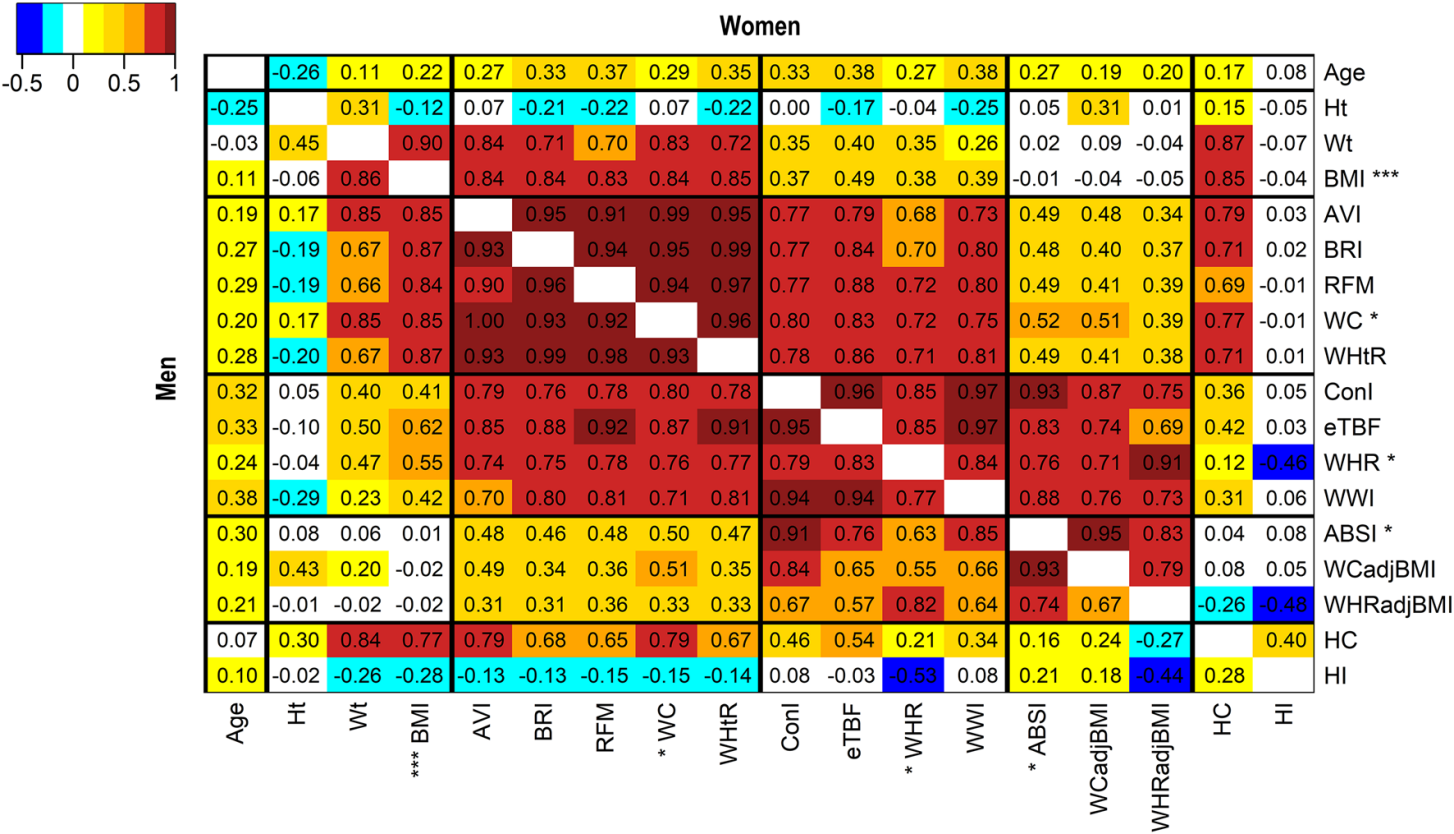
Heatmap of the correlation between anthropometric indices. Age—age at recruitment; AVI—Abdominal Volume Index; ABSI—A Body Shape Index; BMI—Body Mass Index (marked with *** for visibility); BRI—Body Roundness Index; ConI—Conicity Index; eTBF—estimated Total Body Fat; HC—Hip Circumference; HI—Hip Index; RFM—Relative Fat Mass; WC—Waist Circumference; WCadjBMI—WC adjusted for BMI; WHR—Waist-to-Hip Ratio; WHRadjBMI—WHR adjusted for BMI; WHtR—Waist-to-Height Ratio; WWI—Weight-adjusted Waist Index; Cells—partial Pearson correlation coefficients (adjustment for age at recruitment and study centre, except for age at recruitment, which was adjusted only for study centre); Men—bottom-left half; Women—top-right half; *—WC, WHR and ABSI were used as representatives of the strongly, moderately correlated and uncorrelated groups of waist indices in the main analyses (the correlation groups are separated with black lines and indices within them are shown in alphabetical order).

Three groups of waist indices emerged according to the strength of their correlation with BMI: strongly correlated (WC-like, r ≈ 0.85), moderately correlated (WHR-like, r ≈ 0.45) and effectively uncorrelated with BMI (ABSI-like, r ≈ 0) (Fig. 1). All waist indices moderately correlated with BMI included weight in their denominator, except the WHR, which used HC. HC, however, was strongly positively correlated with BMI (r ≈ 0.80) and appears to have acted as a partial adjustment of WC for BMI in the WHR. Waist indices strongly correlated with BMI showed a similar and considerably larger SD increment per 5 kg/m^2^ BMI ( ≈ 1.2 SD in men;  ≈ 0.9 SD in women) compared to ABSI ( ≈ 0.14 SD in men and women) (Table 1).

### Combining BMI with correlated waist indices altered the association patterns with all-cause mortality

**Figure 2:**
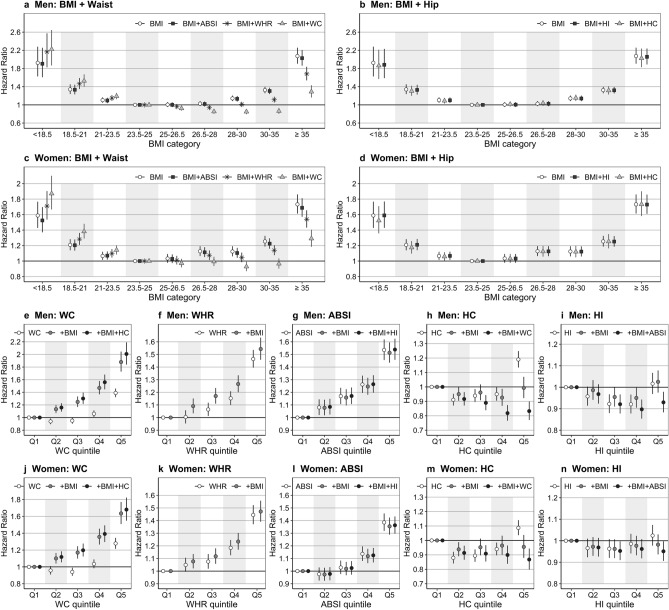
Hazard ratios for the association of obesity indices with all-cause mortality. ABSI—A Body Shape Index; BMI—Body Mass Index; HC—Hip Circumference; HI—Hip Index; WC—Waist Circumference; WHR—Waist-to-Hip Ratio; **a**–**d**—Hazard ratios (points) with 95% confidence intervals (segments) for the association of BMI (reference category 23.5 to < 25 kg/m^2^) with all-cause mortality before and after the addition of waist (**a**,**c**) and hip indices (**b**,**d**) in a delayed-entry Cox proportional hazards model, stratified for age group and study centre and adjusted for smoking status and intensity, attained education level, alcohol intake, physical activity and height (for categorisation of covariates see Supplementary Table S2 online); **e**–**n**—Hazard ratios for the association of waist indices (**e**–**g**,**j**–**l**) or hip indices (h,i,m,n) with all-cause mortality before (white points) and after the addition of BMI (grey points) and a further body-shape index (black points), as indicated in the legends; Q1–5—sex-specific quintile categories (Q1 reference, see cut-offs in Supplementary Table S1 online); Supplementary Fig. S2 online shows plots for the alternative waist indices.

BMI, examined individually, showed a symmetrical U-shaped association with mortality, which was not influenced by adding ABSI or hip indices (Fig. 2a–d). Adding WC, however, shifted the association to a predominantly negative, increasing HRs for low BMI and decreasing HRs for high BMI (Fig. 2a,c). Adding WHR had similar, but more modest influence. WC, examined individually, showed a J-shaped association with mortality. Adding BMI increased the HRs and resulted in a positive association for all quintiles, but further adding HC had little influence (Fig. 2e,j). The association of WHR with mortality was close to linear and adding BMI had lesser influence (Fig. 2f,k). ABSI was positively associated with all-cause mortality for all quintiles in men, but only for the high quintiles in women and was not influenced materially by adding BMI and HI (Fig. 2g,l). Alternative waist indices showed association patterns with all-cause mortality similar to WC, WHR or ABSI, according to the strength of their correlation with BMI (see Supplementary Fig. S2 online).

### Hip indices were weakly negatively associated with all-cause mortality

HC, examined individually, showed a U-shaped association with all-cause mortality, which was almost abolished by adding BMI, but a modest negative association appeared after further adding WC (Fig. 2h,m). The association of HI with mortality was similar to HC, but much weaker, especially in women (Fig. 2i,n). We, therefore, examined further risk stratification only according to waist indices.

### The ability of waist indices to separate high-risk from low-risk individuals within underweight and obese categories was dependent on their correlation with BMI

**Figure 3:**
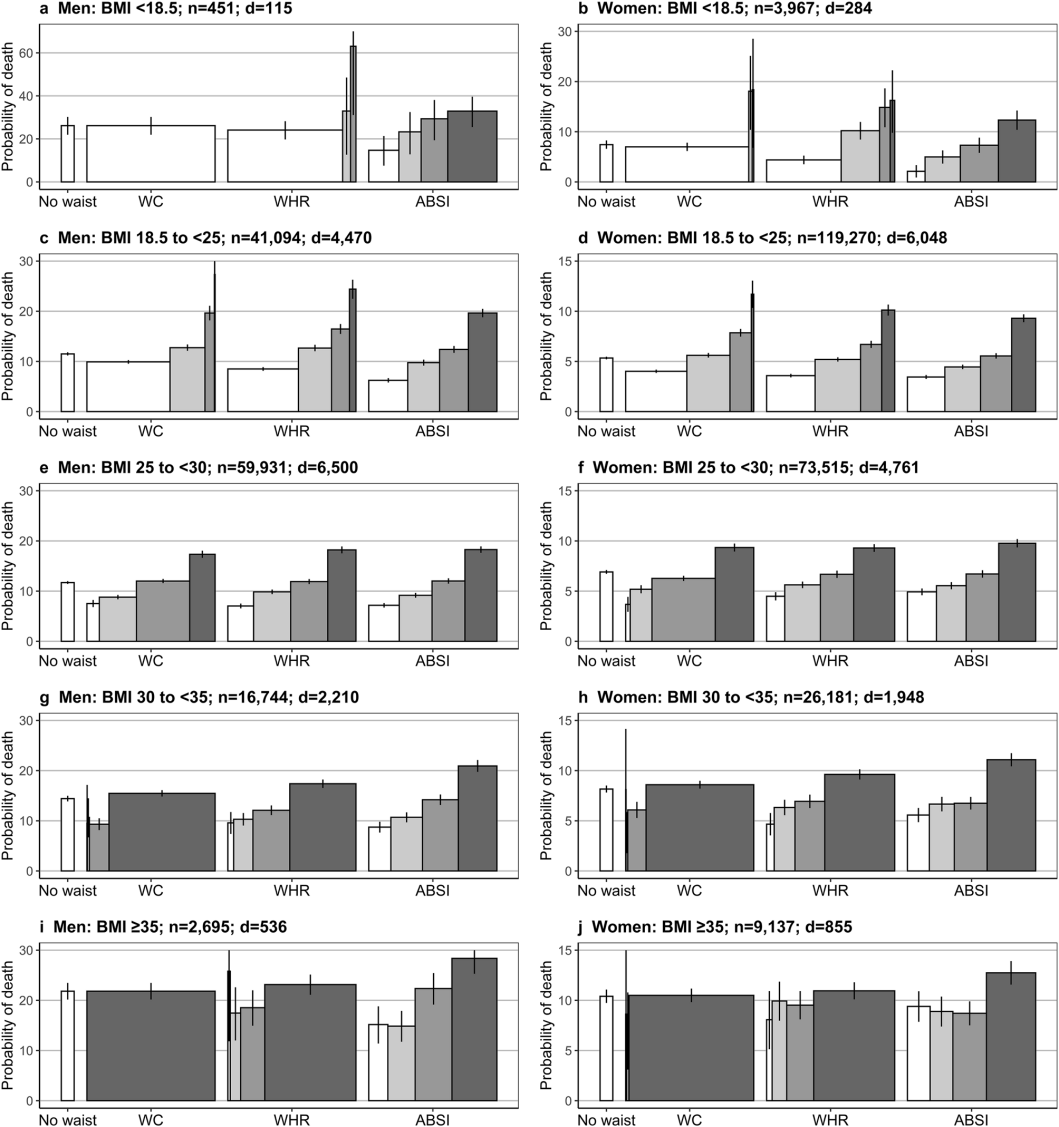
Kaplan–Meier estimates of 15-year probability of death for categories by BMI and waist index. ABSI—A Body Shape Index; BMI—Body Mass Index; WC—Waist Circumference; WHR—Waist-to-Hip Ratio; Waist indices were categorised as sex-specific cohort-wide quartiles (see cut-offs in Supplementary Table S1 online); Bars—the width for waist indices represents the proportion of the individuals in the corresponding waist quartile from the total number of individuals in the corresponding BMI category, colour-coded from white for the lowest to dark for the highest quartile; No waist—probability estimates for the total BMI category, without further stratification according to any waist index; d—number of deaths from all causes recorded during the first 15 years of follow-up per BMI category; n—number of individuals per BMI category; Supplementary Fig. S3 online shows plots for the alternative waist indices.

While the highest sex-specific cohort-wide quartile of all waist indices could separate a high-risk subgroup within the overweight BMI category, including 20% to 27% of men and 29% to 34% of women (see Fig. 3 for Kaplan–Meier estimates of 15-year probability of death within subgroups defined according to BMI and ABSI, WC, or WHR and Supplementary Fig. S3 online for alternative waist indices), most of the underweight and obese individuals belonged to the same quartile of WC-like indices strongly correlated with BMI. On the contrary, every BMI category included sizeable subgroups of all quartiles of ABSI-like indices uncorrelated with BMI. The risk of death was consistently higher in the highest ABSI quartile compared to the other three quartiles, justifying the use of the 75th centile as a cut-off in subsequent analyses. WHR-like indices moderately correlated with BMI showed an intermediate pattern, with small sizes of the low-quartile subgroups among individuals in the obese categories. Although men with WHR in the highest quartile and BMI in the underweight or normal-weight category showed higher mortality compared to men in the overweight or obese BMI category, they represented only a very small proportion of men in the underweight or normal-weight BMI categories.

### Individuals with high-ABSI consistently showed approximately 30% higher risk of death compared to individuals with low-ABSI within each BMI category

The lowest risk of death was in the normal-weight and the overweight subgroups with low-waist when using ABSI, WC with BMI-specific cut-offs, or the WHR (women) to create high-risk and low-risk subgroups, but was in the overweight subgroup with low-waist when using WC with WHO cut-offs or WHR (men) (Table 2). The highest risk of death was in the underweight and obese grade II and III subgroups with high-waist for the three waist indices (ABSI, WC and WHR). The high-WHR and high-WC subgroups were very small (for low BMI) or large (for high BMI), when using WHO cut-offs. On the contrary, the high-ABSI subgroup included 18% to 39% of the individuals within every BMI category and consistently showed 22% to 55% higher risk of death compared to the corresponding low-ABSI subgroup. Although using BMI-specific cut-offs for WC similarly permitted the separation of a sizeable high-risk subgroup within each BMI category, the strong association between WC and BMI was retained. The difference in BMI between high-WC and low-WC subgroups, defined using BMI-specific cut-offs for WC, was more than 1 kg/m^2^ within the normal-weight, overweight, or obese grade I categories, and more that 3 kg/m^2^ within the obese grade II and III category.

**Table 2:** Hazard ratios for all-cause mortality for subgroups defined according to BMI and waist indices.

Men						
BMI	Low-ABSI^a^ < 83.3	High-ABSI^a^ ≥ 83.3	% _High-ABSI_	High/Low-ABSI	*p* value	BMI_High–Low_
< 18.5	1.64 (1.30–2.06)	2.54 (1.99–3.24)	38.6	1.55 (1.12–2.16)	0.009	− 0.20 (0.09)
18.5 to < 25	Reference	1.31 (1.24–1.38)	22.4	1.31 (1.24–1.38)	< 0.0001	− 0.20 (0.02)
25 to < 30	0.98 (0.94–1.02)	1.28 (1.22–1.34)	25.1	1.31 (1.25–1.37)	< 0.0001	0.02 (0.01)
30 to < 35	1.23 (1.16–1.30)	1.59 (1.49–1.70)	30.2	1.29 (1.20–1.39)	< 0.0001	0.07 (0.02)
≥ 35	1.91 (1.72–2.13)	2.46 (2.19–2.76)	35.3	1.29 (1.11–1.49)	0.001	0.10 (0.12)
*p*_interaction_					0.884	

BMI	Low-WHR^b^ < 0.90	High-WHR^b^ ≥ 0.90	% _High-WHR_	High/Low-WHR	*p* value	BMI_High–Low_
< 18.5	1.74 (1.44–2.10)	3.47 (2.38–5.05)	13.3	1.99 (1.32–3.02)	0.001	0.14 (0.11)
18.5 to < 25	Reference	1.14 (1.08–1.20)	52.6	1.14 (1.08–1.20)	< 0.0001	0.78 (0.01)
25 to < 30	0.90 (0.83–0.98)	1.08 (1.03–1.13)	85.4	1.20 (1.11–1.29)	< 0.0001	0.72 (0.02)
30 to < 35	1.34 (1.04–1.73)	1.33 (1.26–1.41)	97.0	1.00 (0.77–1.28)	0.978	0.36 (0.06)
≥ 35	2.44 (1.26–4.71)	2.09 (1.91–2.28)	98.7	0.86 (0.44–1.66)	0.645	− 1.61 (0.48)
*p*_interaction_					0.055	

BMI	Low-WC^b^ < 102 cm	High-WC^b^ ≥ 102 cm	% _High-WC_	High/Low-WC	*p* value	BMI_High–Low_
< 18.5	1.79 (1.51–2.11)	–	0	–	–	–
18.5 to < 25	Reference	1.40 (1.08–1.81)	0.5	1.40 (1.08–1.81)	0.012	1.06 (0.10)
25 to < 30	0.91 (0.88–0.95)	1.20 (1.14–1.26)	18.8	1.31 (1.25–1.38)	< 0.0001	1.46 (0.01)
30 to < 35	0.97 (0.87–1.09)	1.28 (1.22–1.34)	81.8	1.31 (1.17–1.47)	< 0.0001	0.99 (0.03)
≥ 35	–	1.95 (1.80–2.12)	98.8	–	–	–
*p*_interaction_					–	

BMI	Low-WC^c^ < cut-off	High-WC^c^ ≥ cut-off	% _High-WC_	High/Low-WC	*p* value	BMI_High–Low_
< 18.5	1.75 (1.46–2.11)	2.44 (1.63–3.66)	13.7	1.39 (0.89–2.17)	0.144	0.29 (0.11)
18.5 to < 25	Reference	1.10 (1.04–1.16)	27.8	1.10 (1.04–1.16)	0.0007	1.19 (0.02)
25 to < 30	0.92 (0.89–0.96)	1.18 (1.13–1.24)	28.5	1.28 (1.22–1.34)	< 0.0001	1.42 (0.01)
30 to < 35	1.12 (1.05–1.19)	1.52 (1.43–1.62)	33.8	1.36 (1.27–1.46)	< 0.0001	1.24 (0.02)
≥ 35	1.68 (1.52–1.86)	2.82 (2.49–3.20)	27.0	1.68 (1.44–1.96)	< 0.0001	3.36 (0.11)
*p*_interaction_					< 0.0001	

Women						
BMI	Low-ABSI^a^ < 76.2	High-ABSI^a^ ≥ 76.2	% _High-ABSI_	High/Low-ABSI	*p* value	BMI_High–Low_
< 18.5	1.41 (1.23–1.62)	1.87 (1.61–2.17)	28.7	1.33 (1.09–1.62)	0.005	− 0.32 (0.03)
18.5 to < 25	Reference	1.30 (1.24–1.36)	18.4	1.30 (1.24–1.36)	< 0.0001	− 0.04 (0.01)
25 to < 30	1.00 (0.96–1.05)	1.32 (1.25–1.38)	28.5	1.31 (1.25–1.38)	< 0.0001	0.13 (0.01)
30 to < 35	1.11 (1.05–1.18)	1.54 (1.44–1.64)	37.6	1.38 (1.28–1.49)	< 0.0001	0.06 (0.02)
≥ 35	1.63 (1.50–1.78)	1.99 (1.81–2.17)	38.1	1.22 (1.08–1.37)	0.0009	− 0.42 (0.08)
*p*_interaction_					0.473	

BMI	Low-WHR^b^ < 0.85	High-WHR^b^ ≥ 0.85	% _High-WHR_	High/Low-WHR	*p* value	BMI_High–Low_
< 18.5	1.53 (1.38–1.70)	1.52 (1.01–2.31)	2.7	0.99 (0.65–1.52)	0.973	− 0.03 (0.07)
18.5 to < 25	Reference	1.33 (1.25–1.41)	7.7	1.33 (1.25–1.41)	< 0.0001	0.65 (0.02)
25 to < 30	0.99 (0.96–1.03)	1.22 (1.16–1.28)	26.9	1.23 (1.17–1.29)	< 0.0001	0.46 (0.01)
30 to < 35	1.08 (1.01–1.15)	1.38 (1.30–1.46)	48.3	1.28 (1.19–1.38)	< 0.0001	0.23 (0.02)
≥ 35	1.59 (1.44–1.76)	1.78 (1.65–1.93)	56.8	1.12 (0.99–1.26)	0.067	0.15 (0.07)
*p*_interaction_					0.053	

BMI	Low-WC^b^ < 88 cm	High-WC^b^ ≥ 88 cm	% _High-WC_	High/Low-WC	*p* value	BMI_High–Low_
< 18.5	1.48 (1.34–1.64)	–	0.2	–	–	–
18.5 to < 25	Reference	1.33 (1.18–1.50)	1.8	1.33 (1.18–1.50)	< 0.0001	1.13 (0.03)
25 to < 30	0.95 (0.92–0.99)	1.19 (1.14–1.25)	28.9	1.25 (1.19–1.32)	< 0.0001	1.19 (0.01)
30 to < 35	0.98 (0.88–1.09)	1.23 (1.17–1.29)	83.2	1.25 (1.12–1.41)	0.0001	0.89 (0.02)
≥ 35	1.35 (0.90–2.04)	1.66 (1.55–1.77)	97.2	1.22 (0.81–1.85)	0.338	1.14 (0.22)
*p*_interaction_					–	

BMI	Low-WC^c^ < cut-off	High-WC^c^ ≥ cut-off	% _High-WC_	High/Low-WC	*p* value	BMI_High–Low_
< 18.5	1.47 (1.31–1.64)	1.82 (1.46–2.28)	9.6	1.24 (0.97–1.60)	0.086	0.16 (0.04)
18.5 to < 25	Reference	1.16 (1.10–1.22)	14.2	1.16 (1.10–1.22)	< 0.0001	1.41 (0.01)
25 to < 30	1.00 (0.96–1.03)	1.24 (1.18–1.31)	20.4	1.25 (1.18–1.32)	< 0.0001	1.23 (0.01)
30 to < 35	1.18 (1.12–1.24)	1.54 (1.39–1.71)	9.3	1.31 (1.17–1.46)	< 0.0001	1.12 (0.03)
≥ 35	1.55 (1.44–1.67)	2.25 (2.00–2.53)	17.9	1.45 (1.27–1.65)	< 0.0001	4.08 (0.08)
*p*_interaction_					0.018	

ABSI—A Body Shape Index; BMI—Body Mass Index; WC—Waist Circumference; WHR—Waist-to-Hip Ratio.

^a^Cut-offs defined as the 75th sex-specific cohort-wide centile, separating the highest quartile.

^b^Cut-offs recommended by the World Health Organisation^18^.

^c^BMI-specific cut-offs: 80, 90, 100, 110 and 125 cm (men); 70, 80, 90, 105 and 115 cm (women) for the corresponding BMI category^20^; Hazard ratios (HR) (95% confidence interval)—derived from delayed-entry Cox proportional hazards models (stratified by age at recruitment and study centre), including a categorical waist-by-BMI cross-classification variable and adjustment variables for smoking status and intensity, alcohol intake, attained education level, physical activity and height (for the categorisation of covariates see Supplementary Table S2 online); % _high_—percentage of individuals from the corresponding BMI category classified as high-waist; High/Low—HRs for high-waist vs low-waist within each BMI category; *p* value –Wald test for the comparison high-waist vs low-waist within each BMI category; p_interaction_—*p* value for statistical interaction on a multiplicative scale, derived from a likelihood ratio test comparing a model including the cross-classification waist-by-BMI variable with a model including separate variables for BMI (five categories) and a waist index (two categories); BMI_High–Low_—mean BMI difference (standard error) between high-waist and low-waist subgroups, derived from a linear model regressing BMI (continuous scale) on a binary variable for high-waist, with adjustment for age at recruitment and study centre; Subgroups with fewer than 3 deaths (marked with “–”) were excluded from the models and, consequently, no tests for interaction were performed for WC.

### Other risk factors had little influence on the separation of a higher-risk subgroup according to high-ABSI

**Figure 4:**
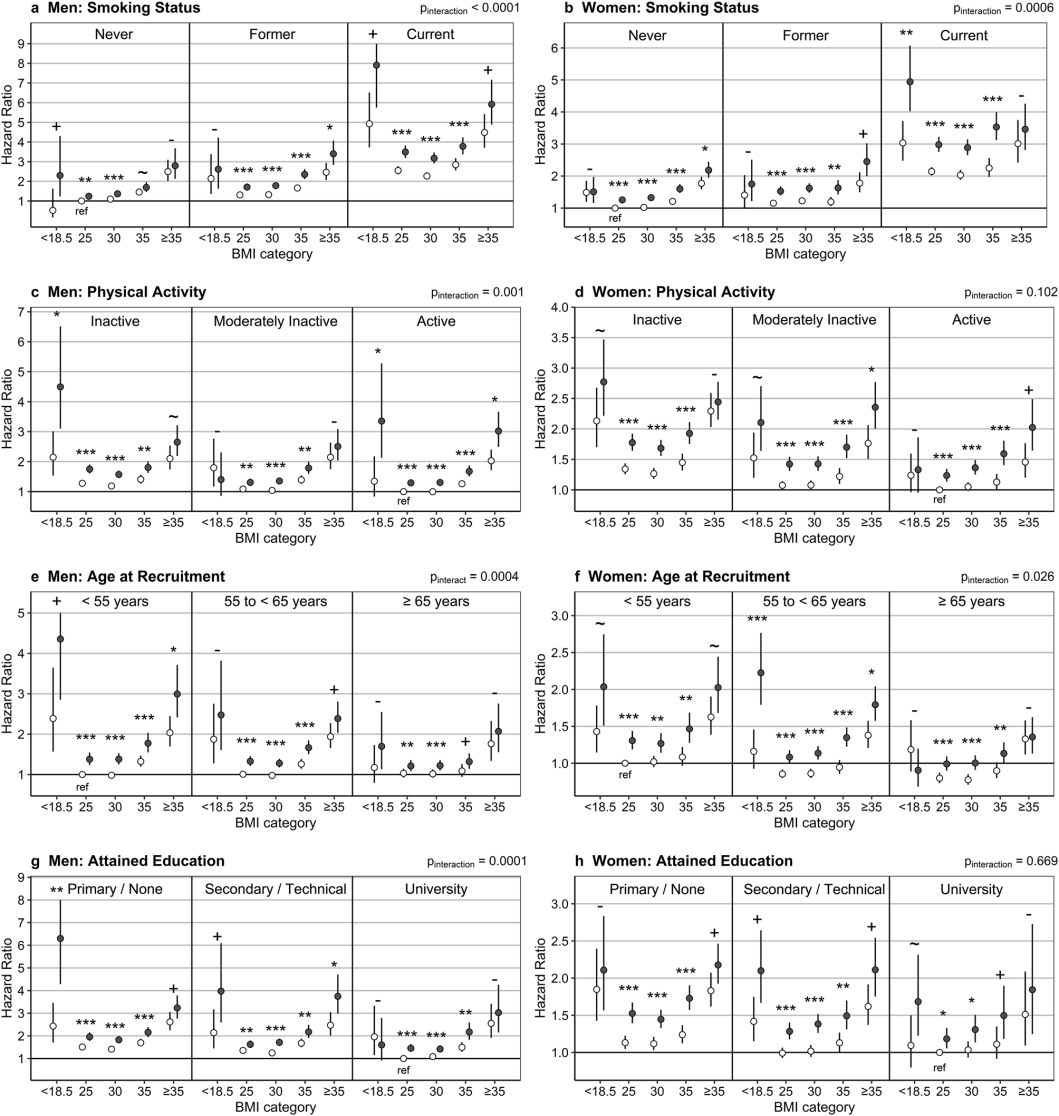
Cross-classification by BMI, ABSI and either smoking status, physical activity, age or attained education. Hazard ratios (points) with 95% confidence intervals (segments)—derived from delayed-entry Cox proportional hazards models, including a cross-classification variable for ABSI-by-BMI-by-factor category (with the “factor” being either smoking status, physical activity, age at recruitment or attained education) and adjustment for smoking status and intensity (omitted for “factor” smoking status), alcohol intake, attained education level (omitted for “factor” attained education), physical activity (omitted for “factor” physical activity) and height and stratified for age at recruitment (omitted for “factor” age) and study centre (for the categorisation of covariates see Supplementary Table S2 online); white points—low-ABSI subgroup: A Body Shape Index (ABSI) < 83.3 for men and < 76.2 for women; dark points—high-ABSI subgroup: ABSI ≥ 83.3 for men and ≥ 76.2 for women; BMI category—body mass index (BMI) category according to the World Health Organisation classification: 25 (18.5 to < 25 kg/m^2^), 30 (25 to < 30 kg/m^2^), 35 (30 to < 35 kg/m^2^); p_interact_—*p* value for statistical interaction on a multiplicative scale, derived from a likelihood ratio test comparing a model including the cross-classification ABSI-by-BMI-by-factor variable with a model including separate variables for ABSI-by-BMI (ten categories) and factor (three categories); *p* values—derived from comparisons of high-ABSI with low-ABSI subgroups within each BMI category: ***p < 0.0001, **p < 0.001, *p < 0.01, + (“plus”) *p* < 0.05, ~ (“tilda”) *p* < 0.1,—(“minus”) *p* ≥ 0.1; hazard ratios (95% confidence intervals) for the latter comparisons are shown in Supplementary Table S4 online.

The risk of death was lowest in the low-ABSI subgroups of normal-weight or overweight individuals for all risk-factor categories by smoking status, physical activity, age at recruitment or attained education (Fig. 4). The high-ABSI subgroup showed approximately 30% higher risk than the corresponding low-ABSI subgroup for BMI 18.5 to 35 kg/m^2^ in most risk-factor categories. There was evidence for effect modification by smoking status, with slightly larger HRs for high-ABSI vs low-ABSI in women current smokers and lower HRs in men never smokers, and by age at recruitment in men, with lower HRs in men aged 65 years or over (see Supplementary Table S4 online).

## Discussion

Absence of adjustment for weight in the calculation of a waist index determined a strong correlation with BMI. Combining BMI with a strongly correlated waist index altered considerably the association patterns with mortality, reversing to a predominantly negative the association with BMI and exaggerating the positive association with the waist index. Combining BMI with a moderately correlated waist index resulted in similar, but more moderate changes. The association patterns were preserved only when combining BMI with waist indices designed to be independent from BMI, such as ABSI. The low-WC and low-WHR subgroups defined using WHO cut-offs included only a limited number of individuals within the obese BMI categories. The high-WC subgroups defined using BMI-specific cut-offs included individuals with considerably higher BMI compared to the corresponding low-WC subgroups, especially within the obese BMI categories, and thus reflected general rather than specifically abdominal obesity. On the contrary, the high-ABSI subgroups had a sensible size and showed higher risk and no major differences in BMI compared to the corresponding low-ABSI subgroups within every BMI category. ABSI and BMI complemented each other, as neither of them could provide individually the risk stratification achieved by their combination. Hip indices were weakly negatively associated with mortality only in combination with BMI and a waist index, in agreement with previous reports^41^, but their practical application for risk stratification appeared limited.

Several studies have described, in agreement with our findings, a J-shaped association of WC with mortality when used in isolation^7, 13, 17, 28, 42^ and a meta-analysis of 18 prospective studies has reported changes in the association patterns of both WC and BMI when combined^17^. Although this meta-analysis had recommended using a waist index in addition to BMI in clinical practice^17^, thus corroborating the conclusions of the earlier EPIC study examining the association of general and abdominal obesity with mortality^7^, subsequent studies have continued to examine waist indices as alternatives rather than as additions to BMI^10, 28, 30^. The large number of alternative waist indices proposed in the literature^10–16, 21^ creates a further confusion and hinders standardisation of the assessment of abdominal adiposity. Our study is, therefore, particularly important because we have demonstrated that combining BMI with any waist index correlated with it will alter the association patterns with mortality and will bias risk estimates to an extent proportional to the strength of the correlation with BMI. The fact that waist indices similarly correlated with BMI showed similar association patterns with all-cause mortality, irrespective of the large differences in their calculation, indicates that this conclusion could be extended further to newly developed waist indices and to other outcomes associated with both abdominal and general obesity. This statistical artefact would also explain the misleading conclusion of risk-prediction models combining BMI and WHR, that individuals with high-waist but normal-weight have higher risk of death than obese individuals^43^. Furthermore, although imaging measures of body fat compartments would undoubtedly provide a superior method of assessment of body composition, an association with body size would similarly affect them. Larger individuals would naturally have larger body compartments, so a direct comparison of untransformed and unadjusted imaging measures would likely encounter a similar problem as the direct comparison of waist circumference measurements.

The strong correlation between WC and BMI is particularly problematic because they both reflect in different ways the same entities: abdominal and peripheral adiposity. They are, therefore, physically related and not simply statistically correlated due to shortcomings of study design, which could be accounted for in a statistical model. This severely limits the variability in the extreme categories, giving them a disproportionately large leverage, which biases mortality risk estimates. Given the strong correlation between WC and BMI, individuals with discordant WC and BMI would be exceptions and they may have features unrelated to obesity, e.g. a high BMI with small WC could be determined by larger lean mass, rather than by fat accumulation. To avoid statistical artefacts driven by the extreme ends of the distributions, a waist index should be operationalised as independent of BMI by design, prior to combining them in a statistical model or using them for cross-classification. Waist indices independent of BMI by design would naturally reflect the additional effect of obesity arising from altered body shape, which is not captured by BMI.

ABSI stands out among the alternative approaches to designing a waist index independent from BMI, because it is based on the allometric principle, which was used to derive BMI^22, 25^. Although the residuals of WC or WHR adjusted for BMI are also independent of BMI by design, they have negative values, unconventional for clinical indices. Further, a population-based dataset would be required to determine the regression coefficients for their calculation, while ABSI can be calculated using the published formula for any isolated individual^22^, i.e. a clinician could calculate ABSI for any patient without the need this patient to be part of a dataset. Furthermore, we have shown that defining multiple BMI-specific cut-offs for WC, as previously proposed^20^, would not be a reliable alternative, as this does not account completely for the strong correlation between WC and BMI, especially in the high BMI categories.

Regarding generalisability, we have demonstrated that ABSI, originally defined in the National Health and Nutrition Examination Survey (NHANES) 1999–2004^22^, was uncorrelated with BMI in the EPIC cohort, likely because of similar weight and height distributions. EPIC women also had comparable adjusted HRs to post-menopausal women from the Women’s Health Initiative cohort: HR = 1.37 (1.28 to 1.47) for the fifth vs. the first quintile^33^. Some differences by ethnicity and sex, however, may exist. ABSI was associated positively with mortality in the Korean National Health Insurance Cohort^13^ and in white and black, but not in Mexican participants in NHANES^22^ or in Japanese women^31^. ABSI has also been criticised for a narrow distribution around the mean, potentially complicating cut-off selections^30^, but we have demonstrated excellent ABSI-based risk stratification in all BMI categories. Another European study has reported optimal ABSI thresholds at 80.7 for men (near the median for EPIC men) and 76.5 for women (near the 75th centile for EPIC women)^28^. Further, a threshold of 83.0 (near the 75th centile for EPIC men) discriminated best sarcopenic obesity among obese individuals with type 2 diabetes^44^.

Enhancing the BMI-based risk stratification with ABSI is potentially useful, as it would outline higher-risk subgroups for closer follow-up and monitoring for metabolic complications. Some combined applications of ABSI with BMI in clinical settings have already been described^45–47^. Although Krakauer & Krakauer have argued that joining ABSI, HI, BMI and height in a combined Anthropometric Risk Indicator (ARI) achieves better risk prediction compared to the individual components^47^, combining ABSI and BMI in a single index would prevent evaluating individually the risks arising from general and abdominal adiposity. The fact that metabolic health can be preserved in obese individuals lacking abdominal adiposity^48, 49^ clearly indicates that the aetiology of obesity extends beyond a perturbed balance of energy intake and expenditure. Studies in animals and humans suggest that alterations in the regulation of the hypothalamic–pituitary–adrenal axis, the peripheral cortisol metabolism and the response to stress are among the outstanding candidates for a mechanistic explanation of the involvement of abdominal adiposity in morbidity and mortality^50–52^. Grading separately the risks arising from general and abdominal adiposity would encourage further research into differentiating their causes and into the development of personalised management strategies targeting specifically abdominal adiposity and not only weight reduction, which would not necessarily improve fat distribution.

Our study has several strength, but also some limitations. We examined a comprehensive list of traditional and non-traditional waist indices in a large prospective cohort, with a long follow-up and a considerable number of deaths, which are major strengths of our study. Anthropometric measures were obtained by trained personnel and were systematically adjusted for clothing, thus avoiding inaccuracies in self-reported values^35^. Our study, however, was limited by the lack of data obtained with imaging techniques, which are considered a gold standard for the assessment of body fat compartments and visceral fat. This precluded evaluation of associations between obesity indices and measures of body composition and fat distribution. Further, there were no laboratory measures of metabolic health or individuals with morbid obesity (BMI > 45 kg/m^2^). There was also no information on sarcopenia or muscle strength to be able to evaluate their potential association with hip indices.

In conclusion, the complex nature of obesity warrants combining indices of general and abdominal adiposity. A waist index should be used to complement and not to replace BMI, as neither of them in isolation reflects adequately the effects of both, body size and body shape. Waist indices unadjusted for weight or BMI by design are correlated strongly with BMI. Combining BMI with a correlated waist index leads to biased and potentially misleading risk estimates and inefficient risk stratification. To avoid statistical artefacts, a waist index should be operationalised as independent of BMI prior to combining both in a statistical model or using them jointly for cross-classification. ABSI, which is independent of BMI by design, complements best BMI and achieves efficient risk stratification in the underweight and obese, as well as in the normal weight and overweight BMI categories.

## Supplementary information

Supplementary file1

## Data Availability

For information on how to submit an application for gaining access to EPIC data and/or biospecimens, please follow the instructions at https://epic.iarc.fr/access/index.php.

## Abbreviations

ABSI: A Body Shape Index
BMI: Body mass index
EPIC: European Prospective Investigation into Cancer and Nutrition
HC: Hip circumference
HI: Hip Index
HR: Hazard ratio
NHANES: National Health and Nutrition Examination Survey
WC: Waist circumference
WHO: World Health Organisation
WHR: Waist-to-hip ratio
