# Supplementary material for: A Body Shape Index (ABSI) achieves better mortality risk stratification than alternative indices of abdominal obesity: results from a large European cohort
Source: Sci Rep. 2020 Sep 3;10:14541. doi: 10.1038/s41598-020-71302-5 (PMC7471961; doi:10.1038/s41598-020-71302-5)
Supplement: Supplementary file 1 — Supplementary file1 [file 41598_2020_71302_MOESM1_ESM.pdf]

ABSI (A Body Shape Index) achieves better mortality risk stratification than alternative indices of abdominal obesity: results from a large European cohort

Christakoudi S, Tsilidis KK, Muller DC, Freisling H, Weiderpass E, Overvad K, Söderberg S, Häggström C, Pischon T, Dahm CC, Zhang J, Tjønneland A, Halkjær J, MacDonald C, Boutron-Ruault MC, Mancini FR, Kühn T, Kaaks R, Schulze MB, Trichopoulou A, Karakatsani A, Peppa E, Masala G, Pala V, Panico S, Tumino R, Sacerdote C, Quirós JR, Agudo A, Sánchez MJ, Cirera L, Barricarte-Gurrea A, Amiano P, Memarian E, Sonestedt E, Bueno-de-Mesquita B, May AM, Khaw KT, Wareham NJ, Tong TYN, Huybrechts I, Noh H, Aglago EK, Ellingjord-Dale M, Ward HA, Aune D, Riboli E

* **corresponding author**: s.christakoudi@imperial.ac.uk

Supplementary Note: Mathematical rational for Body Mass Index and A Body Shape Index 2

Supplementary Fig. S1 Flow-diagram for participants included in the study 4

Supplementary Fig. S2 Hazard ratios for the association of obesity indices with all-cause mortality............. 5

Supplementary Fig. S3 Kaplan-Meier estimates of 15-year probability of death for categories according to BMI and quartiles of alternative waist indices 7

Supplementary Table S1 Centile cut-offs used for the categorisation of anthropometric indices.................... 9

Supplementary Table S2 Covariates by sex and BMI category 10

Supplementary Table S3 BMI of participants who died and those who survived per year 12

Supplementary Table S4 Hazard ratios for high-ABSI compared to low-ABSI in cross-classification by BMI, ABSI and another risk factor 13

Supplementary Note: Mathematical rational for Body Mass Index and A Body Shape Index

In the text below symbols α, β, γ and δ represent regression coefficients (α is the intercept) and numbers in square brackets [ref.] correspond to references in the main document.

A Body Shape Index (ABSI) is the logical complement of Body Mass Index (BMI), because they are both based on the principle of allometry. The main concept of allometry is that mathematical models of the type:

$$Y = \alpha X^{\beta} \quad (1)$$

describe the general rules according to which the size of individual body parts (represented by Y) changes proportional to the change in the overall size of an organism (represented by X) [23, 24].

Model (1) is mathematically equivalent to a log-linear model:

$$\log Y = \log(\alpha) + \beta * \log X \quad (2)$$

Body mass index (BMI) was originally derived from the following model [25]:

$$\log(\text{Weight}) = \log(\alpha) + \beta * \log(\text{Height}) \quad (3)$$

Formula (3) determines the statistical rule according to which weight increases proportional to the increase in body size reflected in height, i.e. the rule determining how weight is scaled with height.

The residuals of model (3), i.e. the part of weight not explained by the theoretical rule, can be derived for each individual as the difference between log of measured weight ($\text{Weight}_{\text{measured}}$) and log of weight predicted by formula (3) for an average individual with the same measured height ($\text{Height}_{\text{measured}}$):

$$\log(\text{Weight}_{\text{measured}}) - \log(\alpha) - \beta * \log(\text{Height}_{\text{measured}}) \quad (4)$$

Taking exponent from (4), to remove the log, and taking into account that: $\exp(\beta * \log(X)) = X^{\beta}$, transforms equation (4) to:

$$\text{Weight}_{\text{measured}} / (\alpha * \text{Height}_{\text{measured}}{}^{\beta}) \quad (5)$$

The familiar formula for BMI can be derived from (5), taking into account that the coefficient β was estimated as 2 in the original study [25] and ignoring the coefficient α, which is constant and does not alter the shape of the association of weight with height:

$$\text{BMI} = \text{Weight} / \text{Height}^{2} \quad (6)$$

BMI is, thus, a relative measure of general obesity, as it is proportional to the ratio of measured weight and weight theoretically predicted for an average individual with the same height.

Krakauer & Krakauer similarly used a log-linear allometric model to determine how waist circumference (WC) is scaled with weight and height in individuals participating in the National Health and Nutrition Examination Survey (NHANES) 1999–2004 [22]. They used a model similar to (3) to determine the theoretical rule describing how WC increases when body size increases due to an increase in weight and/or height:

$$\log(\text{WC}) = \log(\alpha) + \beta * \log(\text{Weight}) + \gamma * \log(\text{Height}) \quad (7)$$

The residuals of model (7), i.e. the part of WC not explained by the theoretical rule, can be derived for each individual as the difference between log of measured WC ($\text{WC}_{\text{measured}}$) and log of WC predicted from formula (7) for an average individual with the same measured weight and height:

$$\log(\text{WC}_{\text{measured}}) - \log(\alpha) - \beta * \log(\text{Weight}_{\text{measured}}) - \gamma * \log(\text{Height}_{\text{measured}})] \quad (8)$$

Taking exponent from formula (8), to remove the log, and taking into account that: exp (β*log(X)

$+ \gamma * \log(Y)) = X^{\beta} * Y^{\gamma}$, gives a formula similar to (5):

$$WC_{measured} / (\alpha * Weight_{measured}{}^{\beta} * Height_{measured}{}^{\gamma}) \quad (9)$$

In the original study defining ABSI [22], the regression coefficients estimated from model (7) jointly for men and women were:

$$\log(WC) = -2.589 + 0.6807 * \log(Weight) - 0.814 * \log(Height) \quad \text{(7) NHANES}$$

We evaluated, for comparison, model (7) in the European Prospective Investigation into Cancer and Nutrition (EPIC), separately for men and women, including also study centre in the equation, and derived similar regression coefficients, i.e. ABSI generalised very well to individuals participating in the EPIC cohort:

$$\log(WC) = -2.612 + 0.6849 * \log(Weight) - 0.860 * \log(Height) \quad \text{(7) EPIC men}$$

$$\log(WC) = -2.648 + 0.6845 * \log(Weight) - 0.895 * \log(Height) \quad \text{(7) EPIC women}$$

Krakauer & Krakauer derived the final formula for ABSI, which is analogous to formula (6) for BMI, by rounding to simple fractions the regression coefficients from model (7) for NHANES and ignoring the intercept α, which does not alter the association of WC with weight and height [22]:

$$ABSI = WC / (Weight^{2/3} * Height^{-5/6}) \quad (10)$$

ABSI is, thus, a relative measure of abdominal obesity, as it is proportional to the ratio of measured WC and WC theoretically expected for an average individual with the same weight and height.

To express ABSI with respect to BMI, the right side of formula (10) can be simultaneously multiplied and divided by $Height^{-4/3}$. Taking into account that a multiplication with $Height^{-4/3}$ is mathematically equivalent to a multiplication with $(Height^{-2})^{2/3}$ and a division by $Height^{-4/3}$ is mathematically equivalent to a multiplication with $Height^{4/3}$ or $Height^{8/6}$:

$$ABSI = WC / [(Weight * Height^{-2})^{2/3} * Height^{8/6} * Height^{-5/6}] \quad (11)$$

Taking into account that $BMI = Weight / Height^{2} = Weight * Height^{-2}$ and consolidating the two terms for Height, results in a formula that illustrates the relationship between ABSI and BMI:

$$ABSI = WC / (BMI^{2/3} * Height^{1/2}) \quad (12)$$

Although formulas (10) and (12) are mathematically equivalent, formula (10) is more appropriate for calculating ABSI, as it uses two measured entities (Weight and Height) and, thus, minimises the rounding error which arises from using a calculated entity such as BMI.

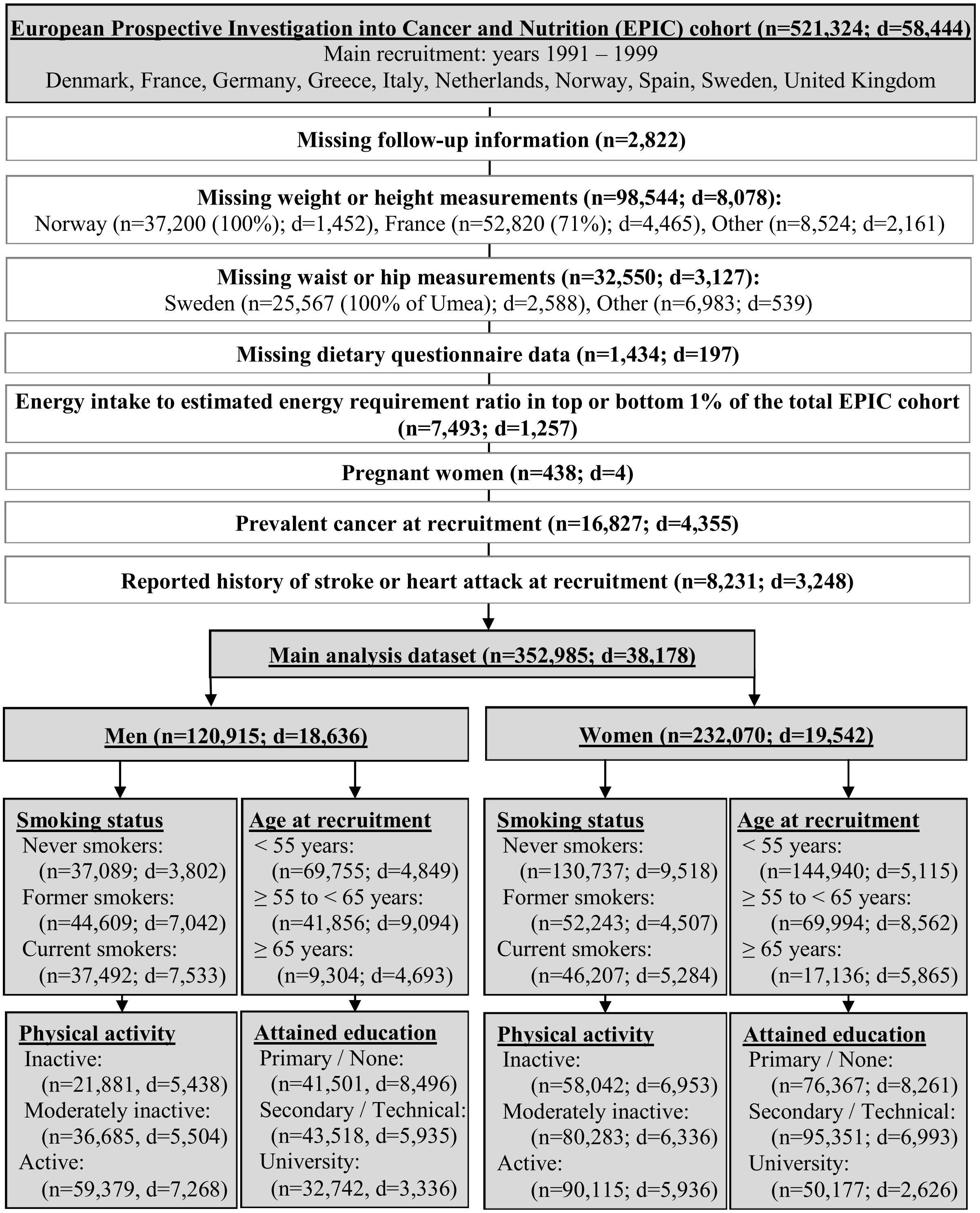

Supplementary Fig. S1 Flow-diagram for participants included in the study

Each step up to the main analysis dataset shows sequential exclusions determined by data availability and quality. The boxes below the main dataset show the subgroups used for cross-classification according to the major risk factors for death, which could also influence obesity; **n** – number of individuals; **d** – number of deaths.

Supplementary Fig. S2 Hazard ratios for the association of obesity indices with all-cause mortality

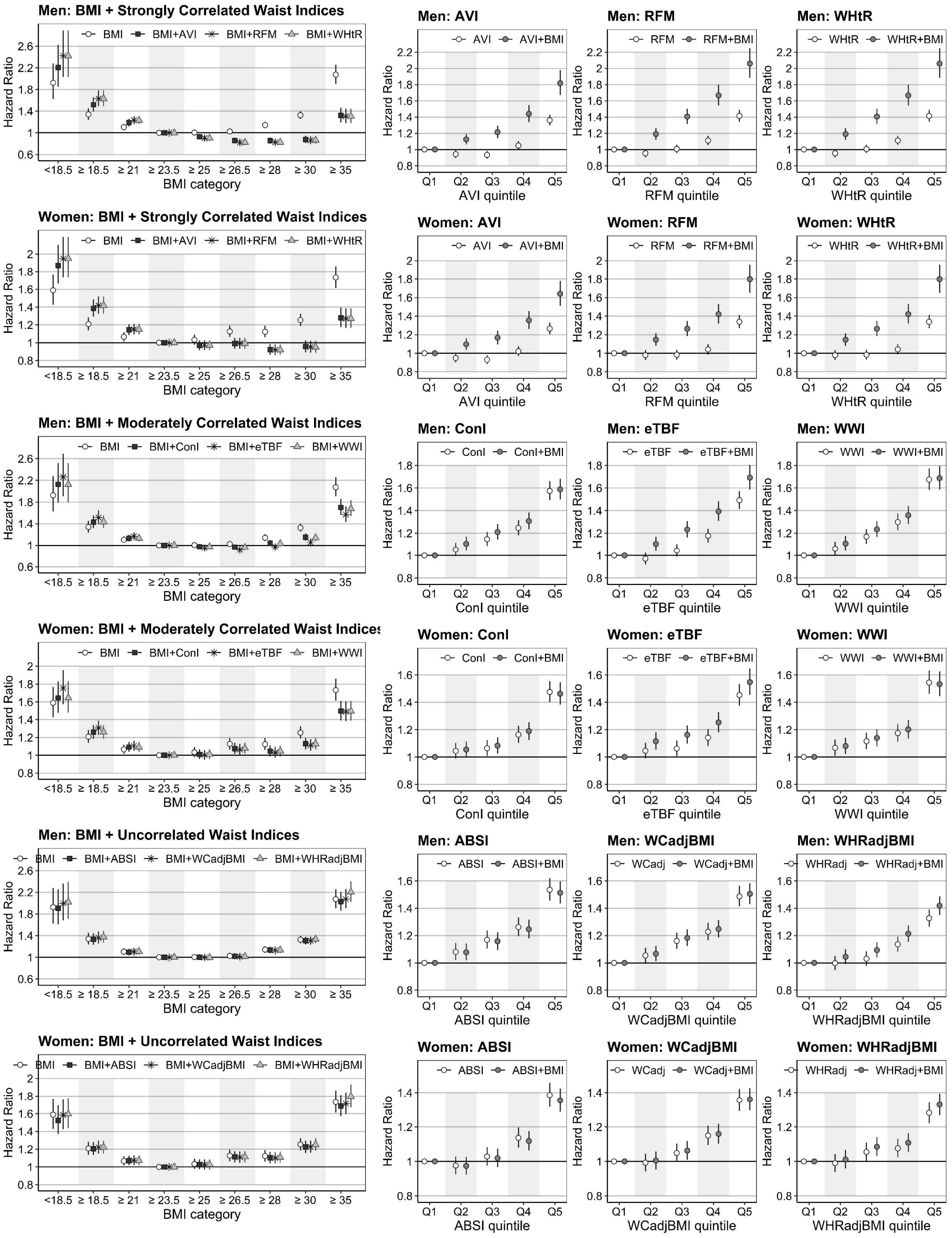

ABSI – A Body Shape Index; **AVI** – Abdominal Volume Index; **BMI** – Body Mass Index; **ConI** – Conicity Index; **eTBF** – estimated Total Body Fat; **RFM** – Relative Fat Mass; **WC** – Waist Circumference; **WCadjBMI** – WC adjusted for BMI; **WHR** – Waist-to-Hip Ratio; **WHRadjBMI** – WHR adjusted for BMI;

WHtR – Waist-to-Height Ratio; **WWI** – Weight-adjusted Waist Index.

First column – hazard ratios (points) with 95% confidence intervals (segments) for the association of BMI (reference category 23.5 to <25 kg/m^2) with all-cause mortality before and after the addition of a waist index in a delayed-entry Cox proportional hazards model, stratified for age group and study centre and adjusted for smoking status and intensity, attained education level, alcohol intake, physical activity and height (for categorisation of adjustment variables see Supplementary Table S2); **Columns two, three, four** – hazard ratios for the association of waist indices with all-cause mortality before (white points) and after the addition of BMI (grey points); **Q1-5** – sex-specific quintile categories (Q1 reference, see cut-offs in Supplementary Table S1).

Supplementary Fig. S3 Kaplan-Meier estimates of 15-year probability of death for categories according to BMI and quartiles of alternative waist indices

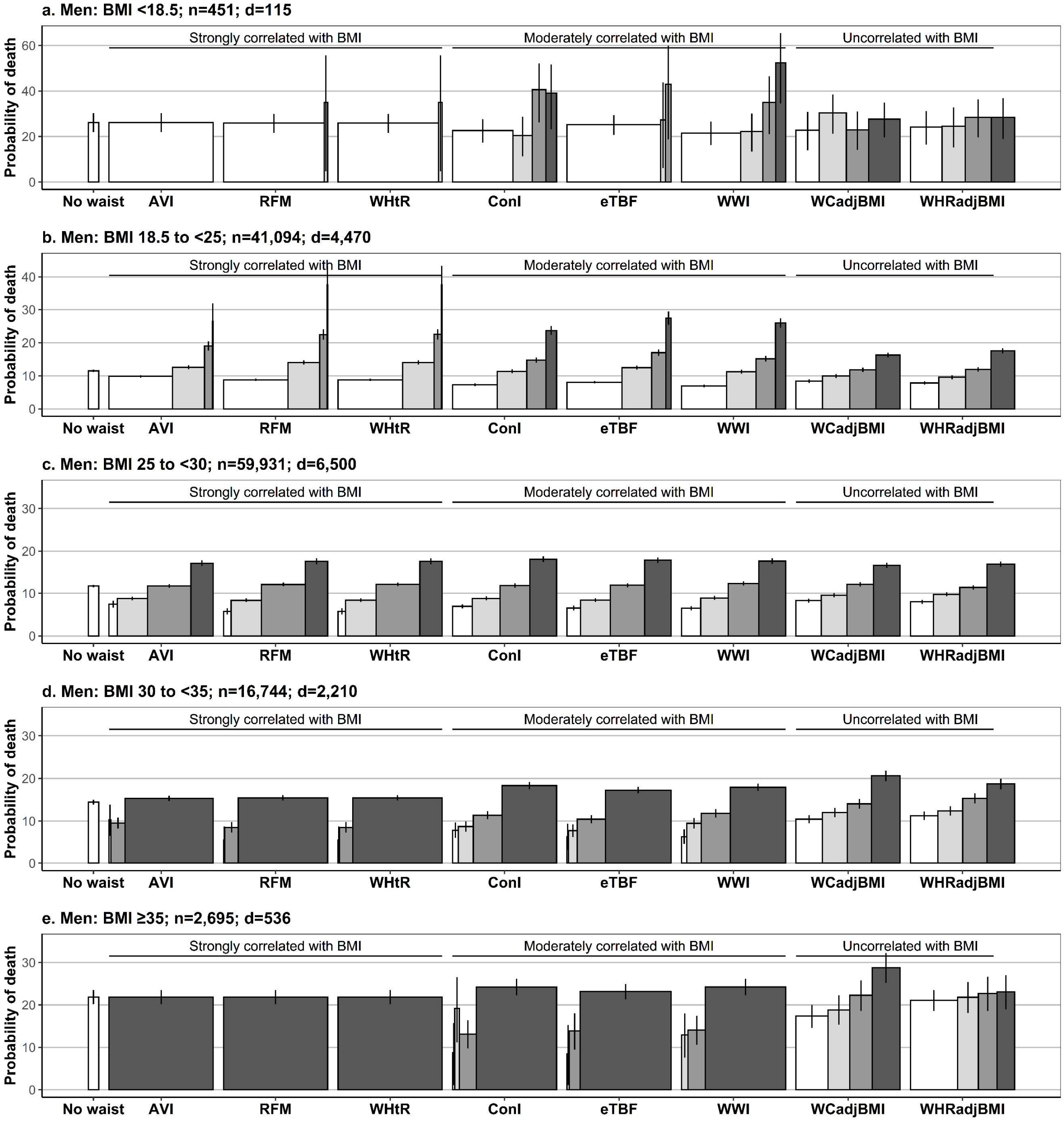

Continues on next page

f. Women: BMI <18.5; n=3,967; d=284

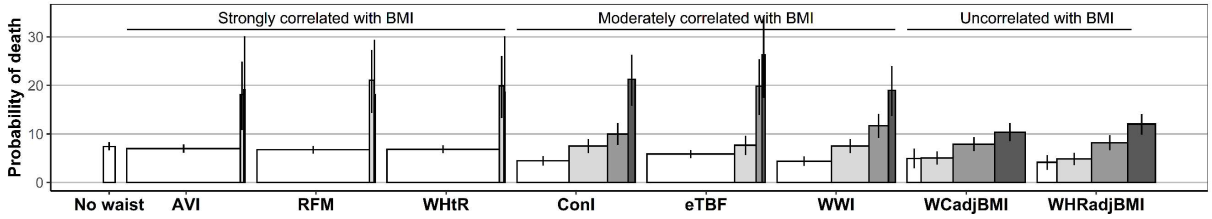

g. Women: BMI 18.5 to <25; n=119,270; d=6,048

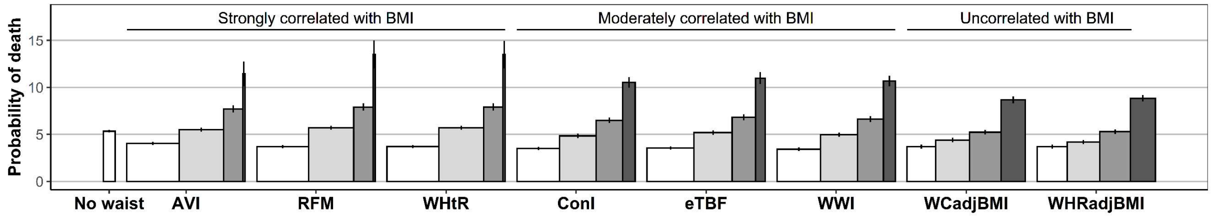

h. Women: BMI 25 to <30; n=73,515; d=4,761

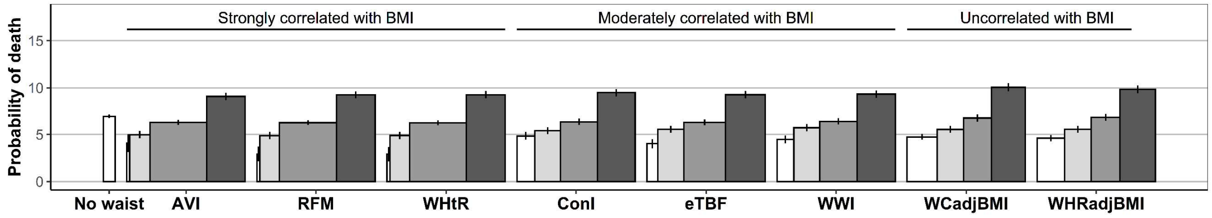

i. Women: BMI 30 to <35; n=26,181; d=1,948

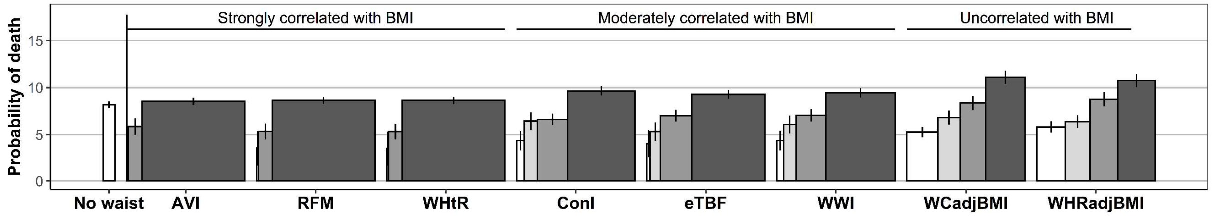

j. Women: BMI ≥35; n=9,137; d=855

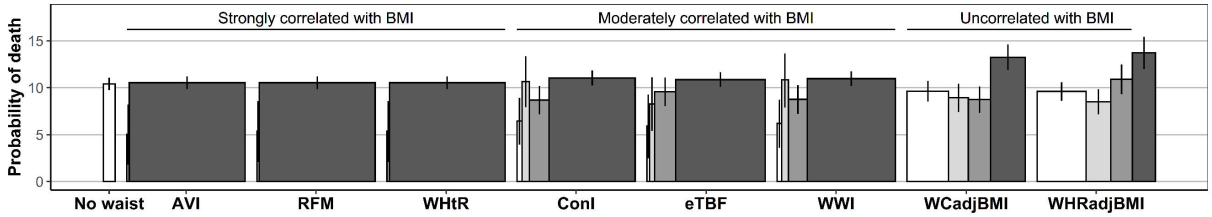

a-e – men; **f-j** – women; **AVI** – Abdominal Volume Index; **BMI** – Body Mass Index; **ConI** – Conicity Index; **eTBF** – estimated Total Body Fat; **RFM** – Relative Fat Mass; **WCadjBMI** – Waist Circumference adjusted for BMI; **WHRadjBMI** – Waist-to-Hip Ratio adjusted for BMI; **WHtR** – Waist-to-Height Ratio; **WWI** – Weight-adjusted Waist Index; Waist indices were categorised using sex-specific cohort-wide quartiles (see cut-offs in Supplementary Table S1); **Bars** – the width for waist indices is proportional to the number of individuals included in the corresponding waist quartile, colour-coded from white for the lowest to dark for the highest quartile; **No waist** – mortality estimates for the total BMI category, without further stratification according to a waist index; **d** – number of deaths recorded during the first 15 years of follow-up per BMI category; **n** – number of individuals per BMI category.

Supplementary Table S1 Centile cut-offs used for the categorisation of anthropometric indices

Centile	ABSI	AVI	BRI	ConI	eTBF	HC	Height	HI	RFM	WC	WCadj	WHR	WHRadj	WHtR	WWI
**Men**															
**Quintiles ***															
**20th**	77.25	14.95	3.203	1.215	17.85	95.1	168.0	0.1402	23.32	86.0	-0.04064	0.89	-0.04043	0.4916	9.985
**40th**	79.63	16.93	3.834	1.258	21.60	99.0	172.5	0.1432	25.88	92.0	-0.0125	0.92	-0.01323	0.5247	10.37
**60th**	81.63	18.77	4.448	1.295	24.65	102.0	176.2	0.1458	27.96	96.8	0.01176	0.95	0.0107	0.5549	10.70
**80th**	83.97	21.22	5.289	1.338	28.11	106.0	180.5	0.1491	30.32	103.0	0.04068	0.99	0.03949	0.5938	11.10
**Quartiles †**															
**25th**	77.93	15.51	3.370	1.228	18.95	96.1	169.5	0.141	24.05	88.0	-0.03283	0.90	-0.03266	0.5006	10.09
**50th**	80.63	17.71	4.131	1.277	23.13	100.1	174.5	0.1445	26.93	94.0	-0.0003419	0.94	-0.001363	0.5396	10.53
**75th**	83.32	20.41	5.034	1.325	27.13	105.0	179.2	0.1481	29.66	101.0	0.03228	0.98	0.03116	0.5824	10.98
**Women**															
**Quintiles ***															
**20th**	68.89	10.33	2.162	1.070	21.07	93.7	156.0	0.1508	29.64	70.2	-0.04335	0.73	-0.04615	0.4314	9.129
**40th**	71.59	11.81	2.741	1.117	25.56	98.0	160.0	0.1543	33.07	75.8	-0.01559	0.77	-0.01668	0.4658	9.562
**60th**	74.01	13.53	3.424	1.162	29.80	102.0	163.2	0.1573	36.28	81.0	0.009409	0.80	0.009503	0.5035	9.980
**80th**	77.05	16.17	4.510	1.221	35.02	108.0	167.5	0.1609	40.15	89.0	0.04161	0.85	0.0429	0.5579	10.53
**Quartiles †**															
**25th**	69.65	10.71	2.307	1.083	22.30	95.0	157.0	0.1518	30.57	72.0	-0.03557	0.74	-0.03779	0.4403	9.246
**50th**	72.79	12.61	3.065	1.139	27.64	100.0	161.6	0.1558	34.68	78.0	-0.003328	0.79	-0.003672	0.4841	9.765
**75th**	76.16	15.34	4.171	1.204	33.51	106.0	166.1	0.1599	39.07	87.0	0.03198	0.83	0.03282	0.5415	10.37

ABSI – A Body Shape Index; **AVI** – Abdominal Volume Index; **BMI** – Body Mass Index; **BRI** – Body Roundness Index; **ConI** – Conicity Index; **eTBF** – estimated Total Body Fat; **RFM** – Relative Fat Mass; **HC** – Hip Circumference (cm); **HI** – Hip Index; **WC** – Waist Circumference (cm); **WCadjBMI** – WC adjusted for BMI; **WHR** – Waist-to-Hip Ratio; **WHRadjBMI** – WHR adjusted for BMI; **WHtR** – Waist-to-Height Ratio; **WWI -** Weight-adjusted-Waist Index; ***** the quintile boundaries were used without rounding the values when included in association and prediction analyses; † the quartile boundaries as shown (rounded to 4 significant digits) were used for cross-classification.

Supplementary Table S2 Covariates by sex and BMI category

Men						
	**Total**	**BMI <18.5**	**18.5 to <25**	**25 to <30**	**30 to <35**	**BMI ≥ 35**
Cohort size: n	120,915	451	41,094	59,931	16,744	2,695
**Smoking category: n (%)**						
never smoker (reference)	37,089 (30.7)	138 (30.6)	14,145 (34.4)	17,635 (29.4)	4,479 (26.7)	692 (25.7)
former (stopped ≥10 years ago)	14,472 (12.0)	16 (3.5)	3,645 (8.9)	7,729 (12.9)	2,655 (15.9)	427 (15.8)
former (stopped <10 years ago)	28,508 (23.6)	70 (15.5)	8,522 (20.7)	15,129 (25.2)	4,139 (24.7)	648 (24.0)
former (stopping time unknown)	1,629 (1.3)	10 (2.2)	552 (1.3)	795 (1.3)	218 (1.3)	54 (2.0)
current (<15 cigarettes/day)	11,341 (9.4)	68 (15.1)	4,394 (10.7)	5,368 (9.0)	1,306 (7.8)	205 (7.6)
current (15 to 24 cigarettes/day)	12,699 (10.5)	89 (19.7)	4,794 (11.7)	5,971 (10.0)	1,616 (9.7)	229 (8.5)
current (≥25 cigarettes/day)	7,040 (5.8)	32 (7.1)	2,195 (5.3)	3,377 (5.6)	1,186 (7.1)	250 (9.3)
current (cigarettes/day unknown)	6,412 (5.3)	25 (5.5)	2,287 (5.6)	3,041 (5.1)	904 (5.4)	155 (5.8)
data missing	1,725 (1.4)	3 (0.7)	560 (1.4)	886 (1.5)	241 (1.4)	35 (1.3)
**Physical activity: n (%)**						
inactive (reference)	21,881 (18.1)	131 (29.0)	6,219 (15.1)	10,945 (18.3)	3,845 (23.0)	741 (27.5)
moderately inactive	36,685 (30.3)	132 (29.3)	12,419 (30.2)	18,319 (30.6)	5,013 (29.9)	802 (29.8)
moderately active	29,205 (24.2)	92 (20.4)	10,104 (24.6)	14,480 (24.2)	3,941 (23.5)	588 (21.8)
active	30,174 (25.0)	77 (17.1)	11,045 (26.9)	14,825 (24.7)	3,691 (22.0)	536 (19.9)
data missing	2,970 (2.5)	19 (4.2)	1,307 (3.2)	1,362 (2.3)	254 (1.5)	28 (1.0)
**Alcohol consumption: n (%)**						
none (reference)	8,065 (6.7)	50 (11.1)	2482 (6.0)	3974 (6.6)	1322 (7.9)	237 (8.8)
0.1 to 4.9 g/day	21,793 (18.0)	120 (26.6)	8,087 (19.7)	10,168 (17.0)	2,865 (17.1)	553 (20.5)
5.0 to 14.9 g/day	31,136 (25.8)	122 (27.1)	11,341 (27.6)	15,232 (25.4)	3,851 (23.0)	590 (21.9)
15.0 to 29.9 g/day	27,283 (22.6)	67 (14.9)	9,401 (22.9)	13,795 (23.0)	3,544 (21.2)	476 (17.7)
≥ 30 g/day	32,638 (27.0)	92 (20.4)	9,783 (23.8)	16,762 (28.0)	5,162 (30.8)	839 (31.1)
**Attained education level [#]: n (%)**						
no school degree (reference)	5,488 (4.5)	13 (2.9)	740 (1.8)	2,760 (4.6)	1,685 (10.1)	290 (10.8)
primary-school degree	36,013 (29.8)	97 (21.5)	9,248 (22.5)	19,021 (31.7)	6,520 (38.9)	1,127 (41.8)
technical/professional degree	29,559 (24.4)	104 (23.1)	10,183 (24.8)	14,982 (25.0)	3,695 (22.1)	595 (22.1)
secondary-school degree	13,959 (11.5)	77 (17.1)	5,647 (13.7)	6,520 (10.9)	1,501 (9.0)	214 (7.9)
university degree	32,742 (27.1)	139 (30.8)	13,997 (34.1)	15,198 (25.4)	2,997 (17.9)	411 (15.3)
data missing	3,154 (2.6)	21 (4.7)	1,279 (3.1)	1,450 (2.4)	346 (2.1)	58 (2.2)
**Height: n (%)**	**Total**	**BMI <18.5**	**18.5 to <25**	**25 to <30**	**30 to <35**	**BMI ≥ 35**
Quintile 1: ≤ 1.680 m	24,269 (20.1)	78 (17.3)	5,930 (14.4)	12,632 (21.1)	4,808 (28.7)	821 (30.5)
Quintile 2: >1.680 to ≤1.725 m	24,383 (20.2)	71 (15.7)	7,360 (17.9)	12,652 (21.1)	3,727 (22.3)	573 (21.3)
Quintile 3: >1.725 to ≤1.762 m	24,075 (19.9)	88 (19.5)	8,161 (19.9)	12,165 (20.3)	3,161 (18.9)	500 (18.6)
Quintile 4: >1.762 to ≤1.805 m	24,261 (20.1)	94 (20.8)	9,299 (22.6)	11,666 (19.5)	2,749 (16.4)	453 (16.8)
Quintile 5: >1.805 m	23,927 (19.8)	120 (26.6)	10,344 (25.2)	10,816 (18.0)	2,299 (13.7)	348 (12.9)

Women						
	**Total**	**BMI <18.5**	**18.5 to <25**	**25 to <30**	**30 to <35**	**BMI ≥ 35**
Cohort size: n	232,070	3,967	119,270	73,515	26,181	9,137
**Smoking category: n (%)**						
never smoker (reference)	130,737 (56.3)	2,166 (54.6)	63,228 (53.0)	42,263 (57.5)	16,990 (64.9)	6,090 (66.7)
former (stopped ≥10 years ago)	17,984 (7.7)	228 (5.7)	9,640 (8.1)	5,697 (7.7)	1,770 (6.8)	649 (7.1)
former (stopped <10 years ago)	32,502 (14.0)	461 (11.6)	17,747 (14.9)	10,251 (13.9)	3,002 (11.5)	1,041 (11.4)
former (stopping time unknown)	1,757 (0.8)	34 (0.9)	964 (0.8)	548 (0.7)	158 (0.6)	53 (0.6)
current (<15 cigarettes/day)	23,924 (10.3)	529 (13.3)	14,030 (11.8)	6,874 (9.4)	1,915 (7.3)	576 (6.3)
current (15 to 24 cigarettes/day)	16,834 (7.3)	406 (10.2)	9,125 (7.7)	5,401 (7.3)	1,480 (5.7)	422 (4.6)
current (≥25 cigarettes/day)	4,134 (1.8)	73 (1.8)	2,130 (1.8)	1,299 (1.8)	460 (1.8)	172 (1.9)
current (cigarettes/day unknown)	1,315 (0.6)	29 (0.7)	836 (0.7)	327 (0.4)	84 (0.3)	39 (0.4)
data missing	2,883 (1.2)	41 (1.0)	1,570 (1.3)	855 (1.2)	322 (1.2)	95 (1.0)

Continues on next page

Supplementary Table S2 (continued)

	Total	BMI <18.5	18.5 to <25	25 to <30	30 to <35	BMI ≥ 35
**Physical activity: n (%)**						
inactive (reference)	58,042 (25.0)	791 (19.9)	21,826 (18.3)	21,016 (28.6)	10,196 (38.9)	4,213 (46.1)
moderately inactive	80,283 (34.6)	1,473 (37.1)	42,647 (35.8)	25,222 (34.3)	8,244 (31.5)	2,697 (29.5)
moderately active	51,027 (22.0)	978 (24.7)	29,774 (25.0)	14,658 (19.9)	4,331 (16.5)	1,286 (14.1)
active	39,088 (16.8)	663 (16.7)	23,046 (19.3)	11,464 (15.6)	3,078 (11.8)	837 (9.2)
data missing	3,630 (1.6)	62 (1.6)	1,977 (1.7)	1,155 (1.6)	332 (1.3)	104 (1.1)
**Alcohol consumption: n (%)**						
none (reference)	38,763 (16.7)	502 (12.7)	13,509 (11.3)	14,302 (19.5)	7,398 (28.3)	3,052 (33.4)
0.1 to 4.9 g/day	85,193 (36.7)	1,632 (41.1)	42,586 (35.7)	26,982 (36.7)	10,168 (38.8)	3,825 (41.9)
5.0 to 14.9 g/day	66,807 (28.8)	1,196 (30.1)	38,658 (32.4)	19,880 (27.0)	5,545 (21.2)	1,528 (16.7)
15.0 to 29.9 g/day	26,717 (11.5)	405 (10.2)	15,834 (13.3)	8,026 (10.9)	1,973 (7.5)	479 (5.2)
≥ 30 g/day	14,590 (6.3)	232 (5.8)	8,683 (7.3)	4,325 (5.9)	1,097 (4.2)	253 (2.8)
**Attained education level: # n (%)**						
no school degree (reference)	14,074 (6.1)	19 (0.5)	1,809 (1.5)	5,723 (7.8)	4,574 (17.5)	1,949 (21.3)
primary-school degree	62,293 (26.8)	471 (11.9)	22,983 (19.3)	24,905 (33.9)	10,293 (39.3)	3,641 (39.8)
technical or professional degree	55,218 (23.8)	837 (21.1)	30,108 (25.2)	17,538 (23.9)	5,085 (19.4)	1,650 (18.1)
secondary-school degree	40,133 (17.3)	950 (23.9)	24,880 (20.9)	10,779 (14.7)	2,696 (10.3)	828 (9.1)
university degree	50,177 (21.6)	1,499 (37.8)	34,006 (28.5)	11,372 (15.5)	2,577 (9.8)	723 (7.9)
data missing	10,175 (4.4)	191 (4.8)	5,484 (4.6)	3,198 (4.4)	956 (3.7)	346 (3.8)
**Height: n (%)**						
Quintile 1: ≤ 1.560 m	49,958 (21.5)	473 (11.9)	17,610 (14.8)	18,827 (25.6)	9,364 (35.8)	3,684 (40.3)
Quintile 2: >1.560 to ≤1.600 m	48,264 (20.8)	711 (17.9)	23,172 (19.4)	16,439 (22.4)	5,878 (22.5)	2,064 (22.6)
Quintile 3: >1.600 to ≤1.632 m	41,385 (17.8)	714 (18.0)	22,075 (18.5)	13,094 (17.8)	4,181 (16.0)	1,321 (14.5)
Quintile 4: >1.632 to ≤1.675 m	47,280 (20.4)	867 (21.9)	27,462 (23.0)	13,803 (18.8)	3,914 (14.9)	1,234 (13.5)
Quintile 5: >1.675 m	45,183 (19.5)	1,202 (30.3)	28,951 (24.3)	11,352 (15.4)	2,844 (10.9)	834 (9.1)

used as the nearest available proxy measure of socioeconomic status; **n (%)** – number of individuals in a given category (% percentage from total cohort in column, men or women).

Supplementary Table S3 BMI of participants who died and those who survived per year

	**Men**							
**Year**	**Count per year**		**Age at recruitment**		**Body Mass Index**			
	**Died**	**Survived**	**Died**	**Survived**	**Died**	**Survived**	**p-value**	**p-value (age)**
**1**	339	120,539	61.4 (8.6)	52.7 (9.6)	26.7 (4.2)	26.6 (3.6)	0.343	0.945
**2**	469	120,014	60.8 (8.2)	52.7 (9.6)	26.8 (4.2)	26.6 (3.6)	0.197	0.798
**3**	580	119,215	60.4 (9.1)	52.7 (9.6)	26.8 (4.2)	26.6 (3.6)	0.113	0.514
**4**	707	117,951	61.0 (8.9)	52.7 (9.5)	27.0 (4.3)	26.6 (3.6)	0.004	0.068
**5**	746	116,862	61.0 (8.9)	52.6 (9.5)	27.0 (4.3)	26.6 (3.6)	0.0002	0.006
**6**	781	115,912	60.2 (8.8)	52.6 (9.5)	27.0 (4.4)	26.6 (3.6)	0.0001	0.012
**7**	926	114,655	60.7 (9.1)	52.5 (9.5)	26.7 (4.1)	26.6 (3.6)	0.146	0.774
**8**	1,047	113,037	60.7 (8.4)	52.5 (9.4)	27.1 (4.2)	26.6 (3.6)	<0.0001	0.0002
**9**	996	111,345	60.5 (8.5)	52.4 (9.4)	27.0 (4.1)	26.6 (3.6)	0.0001	0.014
**10**	1,090	109,624	60.8 (8.7)	52.4 (9.3)	27.1 (4.2)	26.6 (3.6)	<0.0001	0.0001
**11**	1,167	104,929	60.6 (8.3)	52.3 (9.3)	26.9 (4.1)	26.5 (3.6)	<0.0001	0.020
**12**	1,190	98,255	60.2 (8.6)	52.2 (9.3)	26.9 (4.0)	26.5 (3.6)	<0.0001	0.006
**13**	1,232	92,370	59.8 (8.2)	52.1 (9.2)	27.1 (4.2)	26.5 (3.6)	<0.0001	<0.0001
**14**	1,235	88,887	59.7 (8.1)	52.0 (9.2)	26.9 (4.1)	26.4 (3.5)	<0.0001	<0.0001
**15**	1,326	85,462	60.1 (7.8)	52.0 (9.1)	26.7 (3.8)	26.4 (3.5)	<0.0001	0.009

	**Women**							
**Year**	**Count per year**		**Age at recruitment**		**Body Mass Index**			
	**Died**	**Survived**	**Died**	**Survived**	**Died**	**Survived**	**p-value**	**p-value (age)**
**1**	197	231,817	61.1 (11.0)	51.2 (10.5)	26.3 (5.6)	25.5 (4.6)	0.036	0.662
**2**	343	231,382	61.0 (9.4)	51.2 (10.5)	26.5 (5.0)	25.5 (4.6)	0.0005	0.337
**3**	489	230,567	60.0 (9.5)	51.2 (10.4)	26.3 (5.1)	25.5 (4.6)	0.0005	0.401
**4**	531	229,362	60.4 (9.4)	51.1 (10.4)	26.6 (5.3)	25.5 (4.6)	<0.0001	0.081
**5**	640	228,161	60.5 (9.1)	51.1 (10.4)	26.2 (5.0)	25.5 (4.6)	0.0003	0.701
**6**	766	227,160	60.6 (9.6)	51.1 (10.4)	26.2 (5.1)	25.5 (4.6)	<0.0001	0.268
**7**	792	226,089	60.6 (9.8)	51.1 (10.4)	26.4 (5.0)	25.5 (4.6)	<0.0001	0.084
**8**	906	224,346	60.1 (9.7)	51.0 (10.3)	26.5 (5.4)	25.5 (4.6)	<0.0001	0.0009
**9**	1,046	222,189	60.4 (9.7)	51.0 (10.3)	26.6 (5.3)	25.4 (4.6)	<0.0001	<0.0001
**10**	1,100	220,227	60.2 (10.0)	51.0 (10.3)	26.2 (5.0)	25.4 (4.5)	<0.0001	0.090
**11**	1,237	213,042	60.7 (9.4)	50.9 (10.2)	26.5 (5.1)	25.4 (4.5)	<0.0001	0.0003
**12**	1,365	202,449	60.6 (9.2)	50.9 (10.2)	26.5 (5.1)	25.3 (4.5)	<0.0001	<0.0001
**13**	1,442	194,886	60.7 (9.1)	50.9 (10.2)	26.0 (4.8)	25.3 (4.5)	<0.0001	0.127
**14**	1,494	188,614	60.3 (9.1)	50.8 (10.2)	26.1 (5.0)	25.2 (4.4)	<0.0001	0.005
**15**	1,548	179,643	60.6 (9.2)	50.7 (10.2)	26.1 (5.0)	25.2 (4.4)	<0.0001	0.0007

Summaries – mean (standard deviation); **Died** – includes individuals who died during a given year; **Survived** – includes individuals who survived to the end of the corresponding year, which includes individuals who died in subsequent years; **p-value** – Wald test from a linear model regressing BMI on vital status at the end of each year, with adjustment only for study centre; **p-value (age)** – Wald test from a linear model regressing BMI on vital status at the end of each year, with adjustment for study centre and age at recruitment.

Supplementary Table S4 Hazard ratios for high-ABSI compared to low-ABSI in cross-classification by ABSI, BMI and another risk factor

Men									
	Low	High	HR (95% CI)	Low	High	HR (95% CI)	Low	High	HR (95% CI)
**BMI**	**Never smokers**			**Former smokers**			**Current Smokers**		
**< 18.5**	3	10	4.41 (1.21 to 16.05)	19	18	1.22 (0.64 to 2.34)	53	40	1.60 (1.06 to 2.42)
**18.5 to < 25**	850	361	1.24 (1.09 to 1.40)	1,179	687	1.30 (1.18 to 1.43)	1,717	1,117	1.37 (1.27 to 1.48)
**25 to < 30**	1,266	567	1.25 (1.13 to 1.38)	2,215	1,352	1.35 (1.26 to 1.45)	1,894	1,392	1.40 (1.30 to 1.50)
**30 to < 35**	375	223	1.16 (0.99 to 1.37)	722	561	1.42 (1.27 to 1.58)	576	495	1.33 (1.18 to 1.50)
**≥ 35**	92	55	1.12 (0.80 to 1.57)	148	141	1.38 (1.10 to 1.74)	126	123	1.32 (1.03 to 1.69)
**BMI**	**Inactive**			**Moderately Inactive**			**Active**		
**< 18.5**	35	29	2.09 (1.28 to 3.44)	21	16	0.78 (0.41 to 1.50)	17	19	2.49 (1.29 to 4.81)
**18.5 to < 25**	865	846	1.37 (1.24 to 1.51)	1,161	622	1.20 (1.09 to 1.33)	1,676	690	1.29 (1.18 to 1.41)
**25 to < 30**	1,297	1,209	1.32 (1.22 to 1.43)	1,622	991	1.30 (1.20 to 1.41)	2,376	1,092	1.31 (1.22 to 1.40)
**30 to < 35**	455	472	1.28 (1.13 to 1.46)	500	378	1.28 (1.12 to 1.47)	709	426	1.33 (1.18 to 1.50)
**≥ 35**	117	113	1.26 (0.97 to 1.64)	97	96	1.17 (0.88 to 1.55)	152	111	1.49 (1.16 to 1.90)
**BMI**	**< 55 years**			**55 to <65 years**			**≥ 65 years**		
**< 18.5**	22	22	1.82 (1.01 to 3.30)	27	21	1.32 (0.75 to 2.34)	27	25	1.45 (0.84 to 2.50)
**18.5 to < 25**	1,133	422	1.38 (1.23 to 1.54)	1,698	1,023	1.32 (1.22 to 1.43)	953	750	1.18 (1.07 to 1.30)
**25 to < 30**	1,586	622	1.41 (1.28 to 1.55)	2,603	1,760	1.31 (1.23 to 1.39)	1,261	991	1.20 (1.10 to 1.31)
**30 to < 35**	554	270	1.34 (1.16 to 1.55)	863	728	1.32 (1.20 to 1.46)	280	298	1.21 (1.03 to 1.43)
**≥ 35**	127	91	1.47 (1.12 to 1.93)	189	182	1.23 (1.00 to 1.51)	56	52	1.17 (0.80 to 1.72)
**BMI**	**Primary school / None**			**Secondary / Technical school**			**University**		
**< 18.5**	33	27	2.59 (1.55 to 4.33)	26	22	1.86 (1.05 to 3.28)	14	13	0.82 (0.38 to 1.75)
**18.5 to < 25**	1,372	952	1.29 (1.19 to 1.41)	1,409	645	1.20 (1.09 to 1.32)	838	421	1.46 (1.29 to 1.64)
**25 to < 30**	2,281	1,774	1.29 (1.21 to 1.38)	1,859	961	1.37 (1.26 to 1.48)	1,050	497	1.30 (1.17 to 1.45)
**30 to < 35**	849	804	1.27 (1.15 to 1.40)	525	307	1.30 (1.12 to 1.49)	268	150	1.45 (1.19 to 1.77)
**≥ 35**	205	199	1.24 (1.02 to 1.50)	100	81	1.52 (1.13 to 2.03)	50	35	1.18 (0.77 to 1.83)

Women									
	Low	High	HR (95% CI)	Low	High	HR (95% CI)	Low	High	HR (95% CI)
**BMI**	**Never smokers**			**Former smokers**			**Current Smokers**		
**< 18.5**	84	57	1.02 (0.72 to 1.42)	29	30	1.24 (0.75 to 2.07)	97	95	1.63 (1.23 to 2.16)
**18.5 to < 25**	2,565	1,126	1.25 (1.17 to 1.35)	1,405	591	1.33 (1.20 to 1.46)	1,843	899	1.39 (1.28 to 1.51)
**25 to < 30**	2,011	1,298	1.30 (1.21 to 1.40)	1,105	593	1.32 (1.19 to 1.46)	934	706	1.42 (1.29 to 1.57)
**30 to < 35**	819	806	1.33 (1.20 to 1.46)	308	221	1.36 (1.15 to 1.62)	247	286	1.57 (1.32 to 1.86)
**≥ 35**	381	371	1.23 (1.07 to 1.42)	133	92	1.38 (1.06 to 1.80)	83	94	1.15 (0.86 to 1.55)
**BMI**	**Inactive**			**Moderately Inactive**			**Active**		
**< 18.5**	78	82	1.30 (0.95 to 1.78)	69	64	1.38 (0.98 to 1.94)	62	35	1.07 (0.71 to 1.63)
**18.5 to < 25**	1,550	995	1.32 (1.22 to 1.43)	2,039	885	1.32 (1.22 to 1.43)	2,207	731	1.24 (1.14 to 1.35)
**25 to < 30**	1,295	1,102	1.33 (1.22 to 1.44)	1,363	791	1.32 (1.21 to 1.44)	1,362	689	1.30 (1.18 to 1.42)
**30 to < 35**	573	663	1.33 (1.19 to 1.49)	426	366	1.39 (1.21 to 1.60)	370	277	1.41 (1.21 to 1.65)
**≥ 35**	315	300	1.07 (0.91 to 1.25)	171	162	1.34 (1.08 to 1.66)	109	94	1.39 (1.05 to 1.83)
**BMI**	**< 55 years**			**55 to <65 years**			**≥ 65 years**		
**< 18.5**	82	44	1.42 (0.99 to 2.06)	80	88	1.92 (1.42 to 2.60)	49	53	0.76 (0.52 to 1.13)
**18.5 to < 25**	2,075	555	1.31 (1.19 to 1.44)	2,404	1,054	1.27 (1.18 to 1.36)	1,404	1,038	1.24 (1.14 to 1.35)
**25 to < 30**	1,057	453	1.24 (1.11 to 1.39)	1,829	1,240	1.32 (1.23 to 1.42)	1,216	938	1.29 (1.19 to 1.41)
**30 to < 35**	330	231	1.35 (1.14 to 1.60)	623	679	1.43 (1.28 to 1.59)	434	423	1.26 (1.10 to 1.45)
**≥ 35**	169	119	1.25 (0.99 to 1.58)	271	294	1.30 (1.10 to 1.53)	160	150	1.02 (0.81 to 1.28)
**BMI**	**Primary School / None**			**Secondary / Technical School**			**University**		
**< 18.5**	61	46	1.14 (0.78 to 1.67)	96	79	1.48 (1.10 to 2.00)	40	40	1.54 (0.99 to 2.39)
**18.5 to < 25**	1,716	941	1.35 (1.24 to 1.46)	2,496	978	1.29 (1.20 to 1.40)	1,150	402	1.18 (1.05 to 1.33)
**25 to < 30**	1,736	1,382	1.29 (1.20 to 1.39)	1,497	849	1.36 (1.25 to 1.48)	505	239	1.26 (1.08 to 1.48)
**30 to < 35**	741	894	1.40 (1.26 to 1.54)	410	303	1.32 (1.14 to 1.53)	112	74	1.35 (1.00 to 1.81)
**≥ 35**	357	387	1.19 (1.03 to 1.38)	159	126	1.30 (1.03 to 1.65)	38	26	1.22 (0.74 to 2.02)

ABSI – A Body Shape Index; **BMI** – Body Mass Index (categories according to the World Health Organisation); **HR** – hazard ratios (95% confidence interval, **CI**) for high-ABSI vs low-ABSI within each

BMI category (for p-values see Figure 4 in the main document), derived from delayed-entry Cox proportional hazards models, including a cross-classification variable for ABSI-by-BMI-by-factor category, with stratification for age at recruitment and study centre and adjustment for smoking status and intensity, alcohol intake, attained education level, physical activity and height (for categorisation of adjustment variables see Supplementary Table S2); variables used as “factor” (smoking status, physical activity, age at recruitment or attained education) were omitted from the models; **High** – number of deaths within high-ABSI category (ABSI≥83.3 for men, or ABSI≥76.2 for women); **Low** – number of deaths within low-ABSI category (ABSI<83.3 for men, or ABSI<76.2 for women).
